# Solacanin C, a new pregnane steroid from *Solanum torvum* fruits: structural elucidation, potent α-glucosidase inhibition, and *in silico* ADMET profiling

**DOI:** 10.1039/d6ra03305a

**Published:** 2026-08-24

**Authors:** Tho Huu Le, Nguyen Thien Han Le, Phu Hoang Dang, Tung Thanh Phan, Phong Thanh Nguyen, Hai Xuan Nguyen, Truong Nhat Van Do, Mai Thanh Thi Nguyen

**Affiliations:** a Faculty of Chemistry, University of Science Ho Chi Minh City, 227 Nguyen Van Cu Street, Cho Quan Ward Ho Chi Minh City 70000 Vietnam nttmai@hcmus.edu.vn +84-907-426-331; b Vietnam National University Ho Chi Minh City Linh Xuan Ward Ho Chi Minh City 70000 Vietnam; c Research Lab for Drug Discovery and Development, University of Science Ho Chi Minh City Vietnam

## Abstract

Fractionation of the ethyl acetate extract of unripe *Solanum torvum* Swartz fruits led to the isolation of a new pregnane-type steroid, solacanin C (1), together with six known steroidal compounds. Their chemical structures were elucidated using comprehensive spectroscopic analyses, including 1D- and 2D-NMR, FTIR, HRESIMS, and ECD calculations to determine absolute configurations. *In vitro* anti-α-glucosidase evaluation indicated that compounds 1–4 and 6 exhibited potent inhibitory activity (IC_50_; 2.0–12.8 µM), with debenzoylcarpesterol (6) being the most active. Molecular docking simulations with yeast α-glucosidase MAL12 confirmed that these isolated steroids occupy the hydrophobic catalytic cleft, forming key hydrogen bonds with residues Arg312, Arg439, and Asp349. Structure–activity relationship analysis indicated that a free 3β-hydroxyl group and moderate glycosylation enhance potency, while bulky acyl groups or long oligosaccharide chains reduce activity due to steric hindrance. *In silico* ADMET profiling further highlighted the therapeutic potential of the isolated leads. The new steroid solacanin C (1) demonstrated the most balanced medicinal chemistry profile with high drug-likeness (QED) and full compliance with Lipinski's rules, while 6 exhibited superior oral bioavailability *F*_50%_ and high Caco-2 permeability. Both compounds showed low predicted toxicity, suggesting that the compact pregnane scaffold of 1 and the optimized cholestane framework of 6 provide robust foundations for the development of natural α-glucosidase inhibitors in type 2 diabetes management.

## Abstract 2

Fractionation of *S. torvum* fruits gave new steroid solacanin C (1) and six known steroids; 1 had balanced drug-likeness/lipinski compliance, 6 best bioavailability/Caco-2 permeability, low toxicity, as α-glucosidase inhibitors.

## Graphical abstract

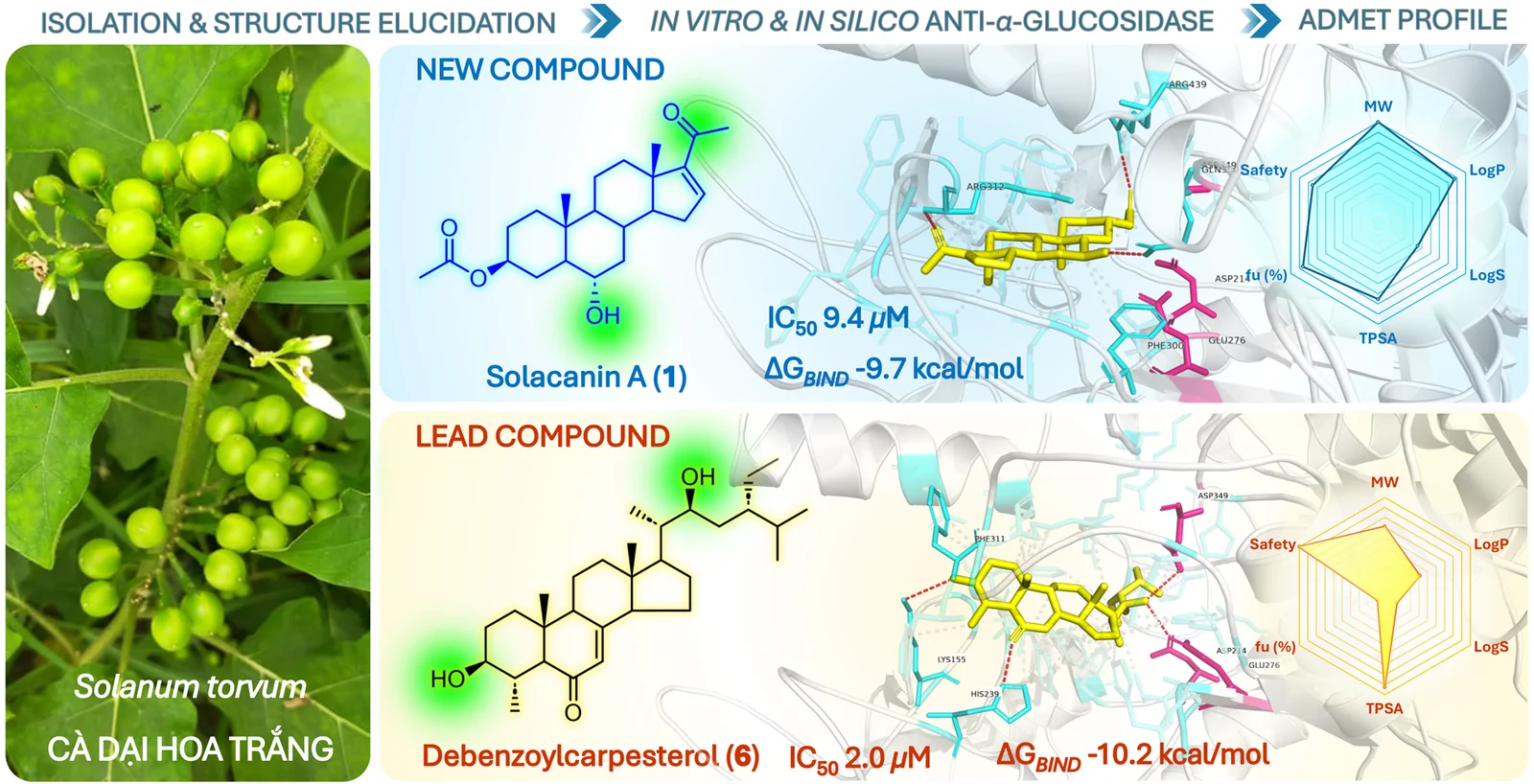

## Introduction

Type 2 diabetes mellitus (T2DM) remains a global health crisis, characterised by chronic hyperglycemia and the subsequent risk of macro- and microvascular complications. A primary therapeutic strategy involves the pharmacological inhibition of α-glucosidase (EC 3.2.1.20), a key intestinal enzyme responsible for the final stage of carbohydrate digestion.^1,2^ By retarding the hydrolysis of oligosaccharides into absorbable glucose, α-glucosidase inhibitors effectively attenuate postprandial glycemic spikes. However, the clinical utility of first-generation α-glucosidase inhibitors, such as acarbose and miglitol, is often compromised by gastrointestinal side effects resulting from the fermentation of undigested sugars.^3,4^ This has catalysed the search for novel, natural product-derived scaffolds that offer potent inhibition with improved tolerability.

*Solanum torvum* Swartz (Solanaceae), commonly known in Vietnam as “Ca dai hoa trang”, is a small woody shrub that typically grows to a height of 2–3 meters.^5^ It is characterised by sparsely spiny stems covered in stellate hairs, alternate ovate leaves with shallow lobes, white flowers that grow in axillary clusters, and spherical berries that turn yellow as they ripen.^6,7^ In traditional practices, unripe green fruits are commonly consumed as vegetables and used as ingredients in curry powder, soups, and sauces. They are also considered functional foods that may help improve blood circulation and enhance immune function in patients highlighting the plant's ethnobotanical significance. Phytochemical investigations have revealed that *S. torvum* possesses a rich chemical profile, including major constituents such as steroidal glycosides,^6,8–12^ steroidal glycoalkaloids,^13^ flavonoids,^11,14^ isoflavonoids,^9^ sesquiterpenes,^15^ and phenolics.^14,16^ Based on these constituents, the plant exhibits a broad spectrum of pharmacological activities, including inhibitory effects on several gastrointestinal cancer cell lines,^12,14^ anti-ulcer activity,^17^ antidiabetic potential,^18,19^ antidepressant effects,^20^ and antibacterial activity.^5,20^

In drug discovery, the yeast α-glucosidase MAL12 from *Saccharomyces cerevisiae* (UniProt P53341) is a well-established surrogate for screening and mechanistic studies. Its catalytic domain features a deep hydrophobic groove guarded by specific residues, including Arg312 and Arg439, which play pivotal roles in substrate recognition and transition-state stabilisation.^21,22^ Understanding how small molecules navigate this narrow gateway and occupy the catalytic cleft is essential for the rational design of more effective inhibitors.^22,23^ Beyond potent inhibitory activity, the clinical utility of α-glucosidase inhibitors is often hampered by suboptimal pharmacokinetic profiles and gastrointestinal side effects. Therefore, evaluating the absorption, distribution, metabolism, excretion, and toxicity (ADMET) properties at an early stage is crucial to ensure that lead compounds possess not only high binding affinity but also favorable drug-likeness and safety margins. While steroidal scaffolds are known for their diverse biological roles, their high lipophilicity often poses challenges for oral bioavailability and off-target interactions.^24^

In this study, we report a systematic investigation of seven steroid-skeleton compounds isolated from the EtOAc of the unripe *S. torvum* fruits. To provide a comprehensive molecular rationale for the observed activities, a dual *in vitro* and *in silico* approach was performed. The experimental inhibitory potencies (IC_50_) were correlated with binding energies and molecular docking poses against the MAL12 receptor. Furthermore, we integrated *in silico* ADMET profiling and drug-likeness analysis (QED and Lipinski's criteria) to evaluate the translational potential of these scaffolds. This integrated analysis allowed us to delineate the SAR of steroid-skeleton scaffolds. Collectively, these results provide insight into the structure–activity relationships of steroid-related scaffolds from *S. torvum* and support their potential as natural α-glucosidase inhibitors for diabetes management.

## Results and discussion

**Fig. 1:**
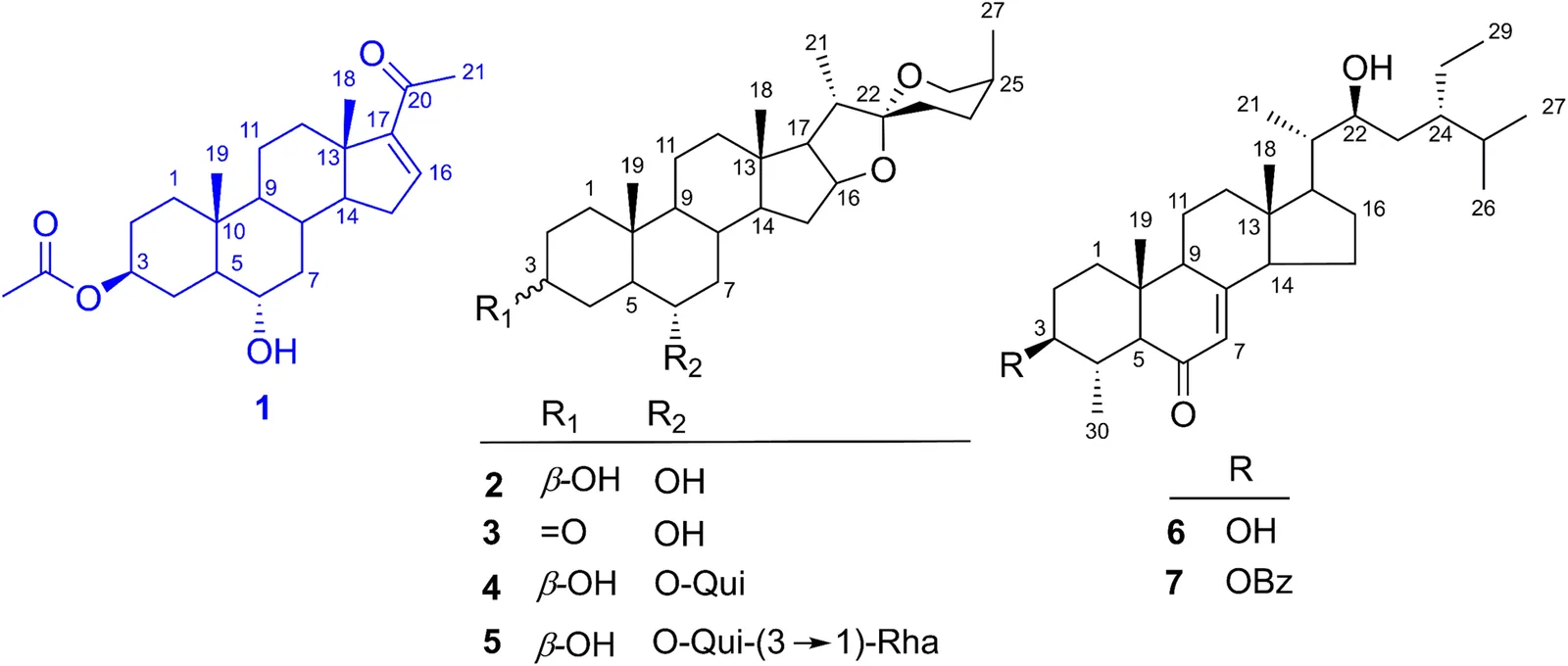
Structures of isolated compounds from unripe *Stovum torvum* fruits.

Unripe *S. torvum* fruits were air-dried and chopped to facilitate efficient extraction. The material was sequentially Soxhlet-extracted with *n*-hexane, EtOAc, and MeOH to yield fractions of varying polarity. Notably, the EtOAc fraction exhibited the most vigorous α-glucosidase inhibitory activity, with an IC_50_ value of 74.5 µg mL^−1^, and was therefore selected for the isolation of bioactive compounds. Using repeated column chromatography and preparative thin-layer chromatography, seven compounds were successfully isolated from the EtOAc extract. Among them, a previously unreported pregnane-type compound, solacanin C (1), were identified. The seven known compounds comprised five spirostan-type compounds, neochlorogenin (2),^25^ neoscorpiogenin (3),^25^ neochlorogenin 6-O-β-d-quinovopyranoside (4),^11^ and neochlorogenin 6-O-α-l-rhamnopyranosyl-(1 → 3)-β-d-quinovopyranoside (5),^11^ as well as two stigmastane-type compounds, namely debenzoylcarpesterol (6)^26^ and carpesterol (7).^27^ The structures of these compounds were elucidated based on comprehensive spectroscopic analyses and comparison with literature data (see Fig. 1 for structures).

**Fig. 2:**
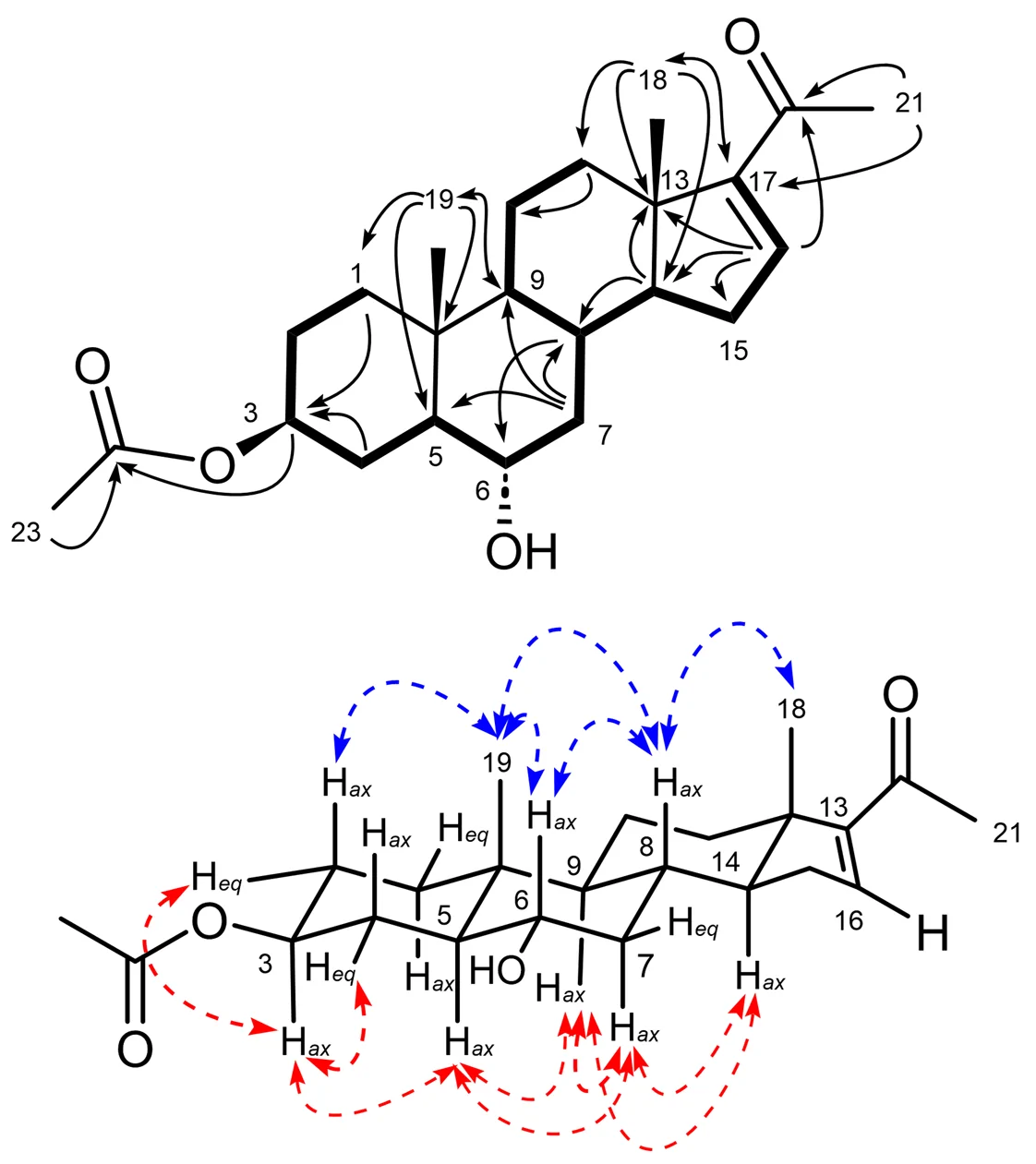
The COSY (bold lines), HMBC (across lines), and NOESY (dashed lines) correlations of compound 1.

Compound 1 was obtained as a white powder and its molecular formula was determined based on HR-ESI-MS analysis, which showed a pseudo-molecular ion peak at *m*/*z* 375.2528 [M + H]^+^ (calcd. for C_23_H_35_O_4_, *m*/*z* 375.2530). The IR spectrum exhibited characteristic absorption bands of *ν*_max_ 3399 cm^−1^ (–OH), 1729 cm^−1^ (C=O), and 1665 cm^−1^ (C=C). The optical rotation was recorded as [*α*]^25^_D_ value of +14.4 (*c* = 0.03 g/100 mL, MeOH). The ^1^H NMR spectrum of 1 showed, in the downfield region, one olefinic proton at *δ*_H_ 6.69 (1H, dd, *J* = 3.8, 2.6 Hz, H-16), consistent with a trisubstituted double bond. In the higher-field region, signals for two oxymethine protons at *δ*_H_ 4.68 (1H, dddd, *J* = 11.5, 9.7, 4.9, 4.8 Hz, H-3) and 3.45 (1H, ddd, *J* = 10.8, 10.7, 4.3 Hz, H-6) were observed, together with four aliphatic methine protons at *δ*_H_ 1.71–0.78. Seven methylene units were identified from multiplets between *δ*_H_ 2.37–0.97, and four methyl groups were evident at *δ*_H_ 2.03 (3H, s, 3-OCOC**H**_3_), 2.25 (3H, s, H-21), 0.88 (3H, s, H-19), and 0.87 (3H, s, H-18). The ^13^C NMR and DEPT spectra indicated 23 carbon signals, comprising two carbonyl carbons at *δ*_C_ 196.9 (C-20) and 170.7(3-O**C**OCH_3_), two olefinic carbons at *δ*_C_ 155.5 (C-17) and 144.3 (C-16), two oxymethine carbons at *δ*_C_ 73.6 (C-3) and 69.5 (C-6), two quaternary sp^3^ carbons at *δ*_C_ 46.4 (C-13) and 36.6 (C-10), four methine carbons at *δ*_C_ 56.1–32.7, seven methylene carbons at *δ*_C_ 41.7–21.1, and four methyl carbons at *δ*_C_ 27.3–13.5 (Table 1). The presence of one ketone carbonyl, one ester carbonyl, one Δ^16,17^ double bond, and a tetracyclic core accounted for the seven degrees of unsaturation and suggested a pregnane-type steroidal framework. Detailed analysis of HSQC and COSY spectra allowed the construction of contiguous spin systems characteristic of a saturated steroidal backbone. In particular, COSY correlations defined fragments from H-1 to H-12, and from H-8–H-14 to H-16. The pregnane skeleton was further corroborated by key HMBC correlations from 19-CH_3_ (*δ*_H_ 0.88) to C-1, C-5, C-9, and C-10 and from 18-CH_3_ (*δ*_H_ 0.87) to C-12, C-13, C-14, and C-17, which are diagnostic for a C_21_ pregnane-type steroid framework. The position of the side-chain carbonyl group was established by HMBC data. The methyl group at *δ*_H_ 2.25 (21-CH_3_) showed long-range correlations with C-20 (*δ*_C_ 196.9) and C-17 (*δ*_C_ 155.5), while H-16 correlated with C-20, indicating that the ketone is at C-20 in a pregnane-21-one-type side chain. The oxymethine at C-3 was deduced from its characteristic chemical shifts (*δ*_H_ 4.68, *δ*_C_ 70.6), together with HMBC cross-peaks from H-1, H-2, and H-4 to C-3. Moreover, the acetate methyl singlet at *δ*_H_ 2.25 (3H, s) exhibited an HMBC correlation to the ester carbonyl at *δ*_C_ 170.7, and H-3 correlated to the same carbonyl, indicating an acetoxy substituent at C-3. Analogously, the oxymethine at C-6 (*δ*_H_ 3.45, *δ*_C_ 69.5) was assigned to a hydroxylated centre based on its deshielded chemical shifts and HMBC correlations from H-5, H-7, and H-8 to C-6, as well as from H-6 to C-7 (Fig. 2). These data collectively established 1 as a 3-acetoxy-6-hydroxy pregnane-21-one bearing a Δ^16,17^ double bond.

**Table 1:** ^1^H-NMR and ^13^C-NMR spectrum data of compound 1 in chloroform-d

Position	*δ*_H_ (*J* in Hz)	*δ*_C_ (*J* in Hz)
1	1.06 (m)	37.0
	1.74 (ddd; 13.3, 10.2, 3.8)	
2	2.00 (m)	32.3
	2.30 (dddd; 17.1, 10.4, 10.6, 3.2)	
3	4.68 (dddd; 11.5, 9.7, 4.9, 4.8)	73.6
4	1.35 (ddd; 17.0, 12.2, 4.3)	34.7
	2.37 (td; 16.5, 12.0)	
5	1.11 (td; 10.4, 3.0)	52.1
6	3.45 (ddd; 10.7, 10.8, 4.3)	69.5
7	0.97 (dd; 11.8, 11.6)	41.7
	2.05 (dd; 4.0, 3.6)	
8	1.71 (dd; 11.2, 3.7)	32.7
9	0.78 (dd; 11.5, 10.3, 4.2)	54.3
10	—	36.6
11	1.39 (dd; 16.8, 12.6, 4.3)	21.1
	1.66 (m)	
12	1.51 (ddd; 14.4, 4.5, 3.3)	27.3
	1.84 (ddd; 13.5, 10.1, 3.8)	
13	—	46.4
14	1.47 (dd; 11.4, 5.0)	56.1
15	1.67 (ddd; 11.4, 3.8)	28.5
	2.22 (ddd; 10.0, 5.0, 2.6)	
16	6.69 (dd; 3.8, 2.6)	144.3
17	—	155.5
18	0.87 (s)	16.0
19	0.88 (s)	13.5
20	—	196.9
21	2.25 (s)	27.3
3-OAc	2.03 (s)	170.7
		21.5

The relative configuration of 1 was elucidated from the NOESY spectrum. Significant NOE correlations of H-3_ax_/H-5_ax_, H-3_ax_/H-4_eq_, H-5_ax_/H-9_ax_, H-5_ax_/H-7_ax_, H-7_ax_/H-9_ax_, H-7_ax_/H-14_ax_, and H-9_ax_/H-14_ax_ indicated that H-3, H-5, H-7, H-9, and H-14 reside on the same molecular face and occupy axial orientations on the steroidal backbone. In contrast, NOESY correlations of H-2_ax_/19-CH_3_, 19-CH_3_/H-8_ax_, 19-CH_3_/H-6_ax_, H-6_ax_/H-8_ax_, and H-8_ax_/18-CH_3_ supported the placement of 18-CH_3_, 19-CH_3_, H-2, H-6, and H-8 on the opposite face. The large vicinal coupling constant of H-3 (*J* = 11.5 Hz) further supported an axial orientation for H-3, consistent with a *trans*-diaxial relationship to neighbouring protons (Fig. 2). Together, these observations established that H-3, H-5, H-6, H-8, and H-14 are axial, whereas the 3-acetoxy and 6-hydroxy substituents adopt equatorial orientations on the steroid skeleton.

**Fig. 3:**
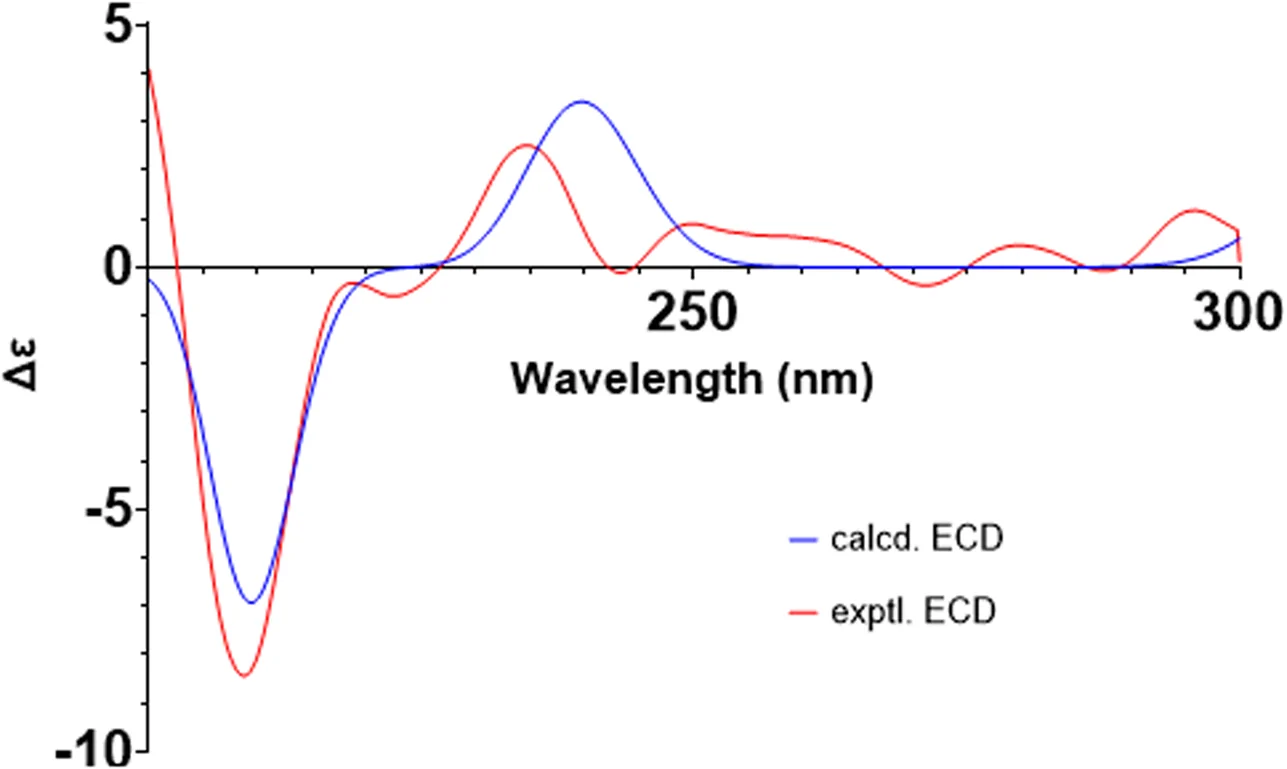
Experimental and calculated ECD spectra of compound 1.

While relative stereochemistry was assigned *via* NMR data, the absolute configuration of 1 required confirmation through ECD analysis for eight stereocenters (C-3, C-5, C-6, C-8, C-9, C-10, C-13, and C-14). Calculated spectra were generated using TDDFT at the CAM-B3LYP/aug-cc-pVDZ/PCM level and compared with experimental data. As shown in Fig. 3, the experimental spectrum exhibited a negative Cotton effect at 208 nm, followed by a positive cotton effect at 234 nm. This characteristic profile was in good agreement with the calculated data for the (3*S*,5*S*,6*S*,8*R*,9*S*,10*R*,13*S*,14*S*)-1 isomer, thereby confirming the absolute configuration. This new metabolite has been designated solacanin C.

**Fig. 4:**
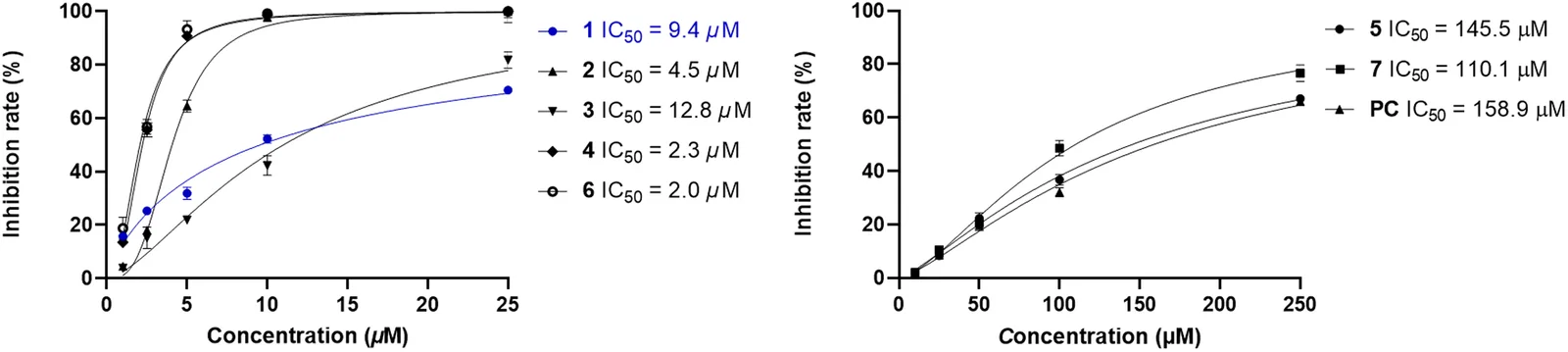
α-Glucosidase inhibitory activities of compounds 1–7 isolated from *S. torvum*.

All compounds isolated from unripe fruits of *S. torvum* were evaluated for their α-glucosidase inhibitory activities at concentrations ranging from 250 to 1 µM. Acarbose, a well-known α-glucosidase inhibitor clinically used for the management of type 2 diabetes, was employed as the positive control. All tested compounds exhibited measurable α-glucosidase inhibition, and, notably, each compound was more than acarbose (IC_50_ = 158.9 µM). Among them, compounds 1–4 and 6 showed strong activity, with IC_50_ values of 9.4, 4.2, 4.5, 12.8, 2.3, and 2.0 µM, respectively, whereas compounds 5 and 7 showed only moderate inhibition, with IC_50_ values of 187.2 and 112.2 µM, respectively (Table S1 and Fig. 4).

To rationalize the *in vitro* α-glucosidase inhibitory activities at the atomic level, molecular docking simulations were performed against the homology model of *Saccharomyces cerevisiae* MAL12 α-glucosidase (UniProt ID: P53341). The computed binding score ranged from −8.2 to −10.2 kcal mol^−1^, exhibiting a strong correlation with the experimental IC_50_ values. The binding score followed the rank order 6 (−10.2) > 3 (−9.9) > 1 (−9.7) > 2 (−9.6) > 7 (−9.2) > 4 (−8.5) > 5 (−8.2) kcal mol^−1^ (Table 2). For reference, acarbose gave a binding score of −8.9 kcal mol^−1^ under the same docking conditions (Table 2 and Fig. S34). All seven isolated compounds showed more favorable binding scores than acarbose, supporting the potential as more potential inhibitor.

**Table 2:** Molecular docking interaction of steroid derivatives (1–7) and α-glucosidase MAL12

Compound	Binding score (kcal mol^−1^)	Type of interaction	Key amino acid
1	−9.7	Hydrogen bonds	Arg312, Arg439, Gln350
2	−9.6	Hydrogen bonds	Glu276, Asp349, Arg439
		π–sigma interaction	His279
		π–alkyl interaction	His239
3	−9.9	Hydrogen bonds	Glu276, His279
		π–alkyl interaction	Pro309
4	−8.5	Hydrogen bonds	His239, Pro309, Phe310
		π–alkyl interaction	Phe157, His245
		Alkyl–alkyl interaction	Leu218, Ala278
5	−8.2	Hydrogen bonds	Arg312, Asp241, Asp349
		Unfavorable interaction	Arg312
		π–sigma interaction	His279
		π–alkyl interaction	His245
6	−10.2	Hydrogen bonds	His239
		Unfavorable interaction	Lys155
		π–sigma interaction	Tyr71, Phe177
		π–alkyl interaction	Phe157
		Alkyl–alkyl interaction	Ala278
7	−9.2	π–sigma interaction	Pro309, Gln350, Arg439
		Unfavorable interaction	Asp349
		π–π stacked interaction	Phe177
		π–alkyl interaction	His239, Phe311
		Alkyl–alkyl interaction	Lys155, Pro240
Acarbose	−8.9	Hydrogen bonds	Asp68, Arg439, Arg212, Phe300, His279, Glu304, Phe311
		Unfavorable donor–donor	His348
		Salt bridge	Glu276, Asp349

**Fig. 5:**
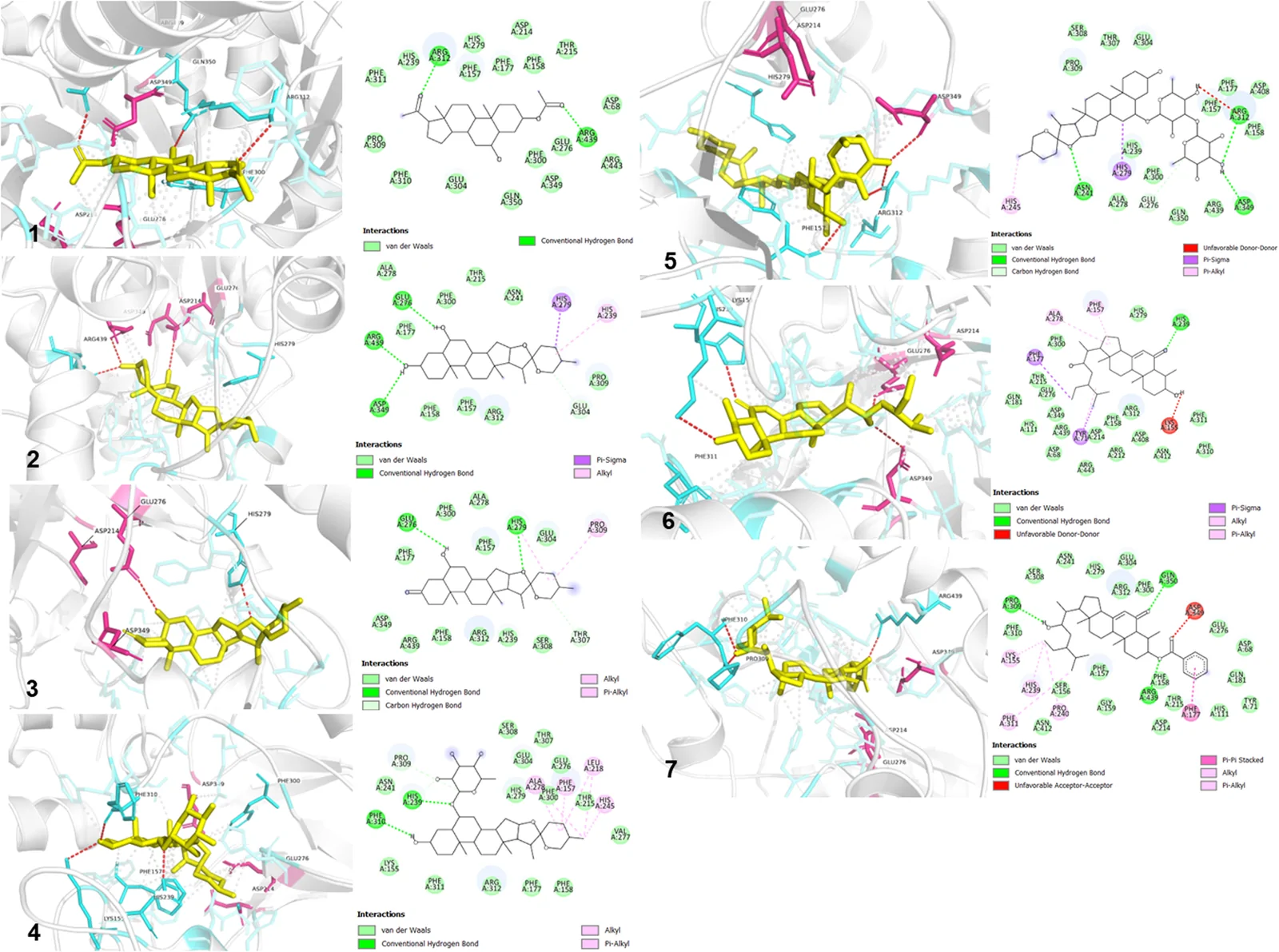
3D docking poses and 2D ligand–protein interaction diagrams of α-glucosidase in complex with compounds 1–7. In 3D diagrams, hydrogen bonds (H-bonds) are depicted as red dashed lines; amino acid residues (aa) of the catalytic triad at the active site are shown in pink, and amino acid residues involved in hydrophobic interactions are shown in cyan.

Among the tested compounds, debenzoylcarpesterol (6) displayed the most potent inhibition (IC_50_ = 2.0 µM; binding score −10.2 kcal mol^−1^). Its superior efficacy was attributed to the optimal three-dimensional architecture of the cholestane skeleton, specifically the extended C-24 ethyl side chain, which facilitates deep penetration into the hydrophobic active site groove. The Δ^7^ double bond and 6-ketone moiety provide a conformational rigidity that likely pre-organized the inhibitor for binding. Although, the 3-hydroxy group showed an unfavorable interaction with Lys155 but the overall structure was allowing 6 to retain potent inhibitory activity (Fig. 5).

A key SAR trend was indicated that the esterification of the 3β-hydroxyl group with a bulky benzoyl moiety resulted in a drastic 55-fold reduction in potency with an IC_50_ value from 2.0 µM to 110.1 µM in compound 7. This loss of activity was two-fold. First, benzoylation eliminated the key hydrogen bond donor capability of the 3-OH group, disrupting essential electrostatic interactions with Asp349 and Glu276. Second, the bulky benzoyl substituent introduced severe steric occlusion, preventing the steroid core from achieving the optimal depth within the catalytic cleft. Docking analysis of 7 revealed an unfavourable interaction with Asp349 and a reliance on weaker π-interactions, which are insufficient to compensate for the loss of the polar anchor (Fig. 5). This pattern aligned well with literature reported that bulky acyl substituents at C-3 of phytosterols or steroidal saponins can mask key hydrogen-bond donors and introduced steric hindrance at the entrance region of the catalytic pocket, thereby weakening enzyme binding.^28,29^

In the spirostane series, glycosylation at the 3β-hydroxy group of neochlorogenin (2) had a pronounced influence on α-glucosidase inhibition. Conversion of the aglycone (2; IC_50_ = 4.5 µM) into its corresponding 3-O-monoglucoside (4; IC_50_ = 2.3 µM) led to an approximately two-fold increase in potency. In contrast, further elongation and branching of the sugar chain, as exemplified by 5 (IC_50_ = 145.5 µM), resulted in a dramatic loss of activity compared with 4 (4 ≫ 5). A molecular docking study showed that the aglycone neochlorogenin (2) formed a hydrogen-bond network with the catalytic residues Glu276 and Asp349. It also suggested that the single quinovose unit of 4 occupying a secondary binding pocket, forming additional stabilising hydrogen bonds with His239, Pro309, and Phe310 without introducing steric clashes. Besides, excessive glycosylation proved deleterious *via* the diglycoside 5 bearing a bulky α-l-rhamnopyranosyl-(1 → 3)-β-d-quinovopyranosyl moiety, showed a dramatic loss of activity with IC_50_ value of 145.5 µM. Although compound 5 retained hydrogen bonds with Asp349 and Asn241, this clash occurred directly at the entrance of the catalytic pocket and appeared to dominate over the retained interactions, in a pattern analogous of compound 7. The disruption of a single consequential interaction at Asp349 outweighed several favorable interactions. This precipitous decline is attributed to steric hindrance imposed by the disaccharide chain at the entrance of the active site. Docking revealed an unfavourable steric clash between the terminal rhamnose unit and Arg312 (Fig. 5). These findings align with established structural insights into GH31 family enzymes, where the narrow “entrance channel” strictly limits the size of ligand substituents, rendering bulky glycosides ineffective.^30^ These data provided appropriately sized sugar chain at C-3 is beneficial for α-glucosidase inhibition, whereas excessive glycosylation becomes detrimental due to steric congestion and suboptimal positioning of the steroidal core within the active site.^28,29,31^ On the other hand, related study on yuccagenin saponins reported the opposite trend with the diglycoside outperformed both the aglycone and the monoglucoside.^32^ This indicated that sugar-chain length alone did not determine inhibitory potency and the sugar type, or attachment position were also decisive. The glycosidic linkage in yuccagenin saponin that study (1 → 4) between rhamnose and glucose, differing from the (1 → 3) linkage in compound 5, which may partly account for the divergent trend. Morever, with docking offering molecular-level insight into which specific residues were subject to steric clash within the binding pocket.

The newly isolated pregnane, solacanin C (1), exhibited significant potency (IC_50_ = 9.4 µM). Although less potent than the optimized cholestane (6) due to a shorter C-21 side chain, 1 achieves a high binding score (−9.7 kcal mol^−1^) through a distinct mechanism. The docking pose indicated that 1 deeply occupies the hydrophobic pocket, while its polar functional groups form critical hydrogen bonds with Arg312 and Arg439, both located near the entrance of the catalytic cleft (Fig. 5). Unlike the bulky glycoside 5, which clashes with this region, the compact scaffold of 1 optimally engages these residues, potentially blocking substrate entry *via* an electrostatic “capping” mechanism.

While neoscorpiogenin (3) showed a high theoretical binding score (−9.9 kcal mol^−1^), the *in vitro* potency (IC_50_ = 12.8 µM) was lower than that of the 3-hydroxy analogue (2). This is rationalized by the electronic properties of the C-3 substituent. The 3-ketone in 3 did not formed interaction with Asp349 observed for 2, relying instead on contacts with Glu276 and His279 (Fig. 5), highlighting the importance of a hydrogen-bond donor at C-3 for optimal engagement with the catalytic residues.

The predicted ADMET profiles for the seven compounds, obtained using the ADMETlab 3.0 platform, are presented in Table 3. The steroidal compounds exhibited diverse pharmacokinetic characteristics, reflecting their structural complexity, with molecular weights ranging from 374.25 to 724.44 Da, lipophilicity (log *P* 2.66–6.13), and variable polarity (TPSA 55.76–176.76 Å2). These features often challenge drug-likeness according to Lipinski-like criteria, particularly the high molecular weight and excessive glycosylation of compound 5 (MW 724.44 Da, TPSA 176.76 Å2), which significantly challenge its membrane permeability and oral bioavailability.

**Table 3:** Predicted physicochemical, drug-likeness, and ADMET profiles of compounds 1–7 (cells are color-coded: green for low risk, yellow for medium risk, and red for high risk)^a^

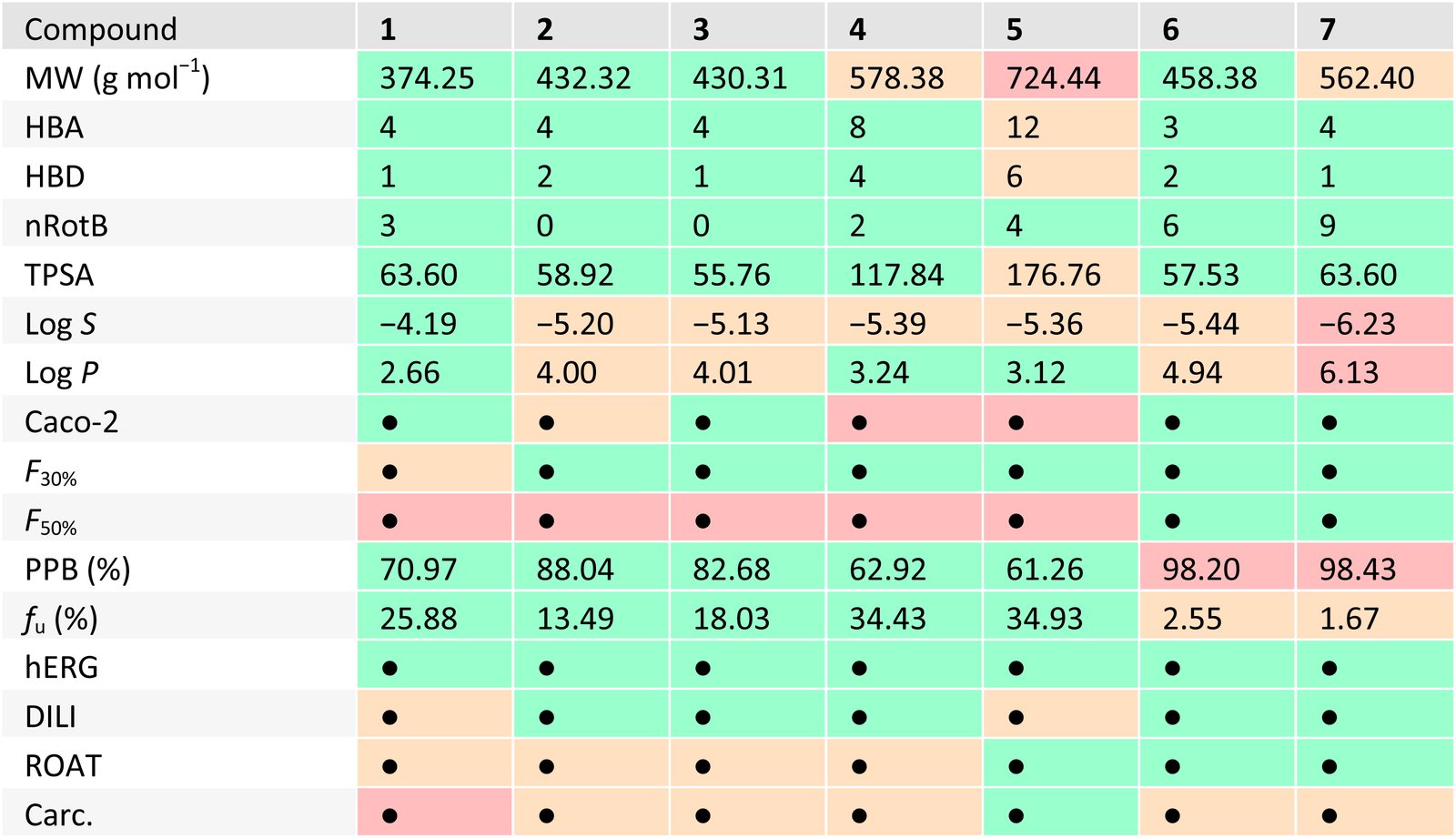

a Risk Assessment: ● low risk (favorable properties, high probability of safety/absorption); ● medium risk (moderate parameters, requires monitoring); ● high risk (potential pharmacokinetic or toxicity liabilities).

*In silico* ADMET profiling highlights a clear distinction between the isolated steroids, with compounds 1 and 6 emerging as the most promising leads. Solacanin C (1) exhibited a superior medicinal chemistry profile, characterized by optimal drug-likeness (MW 374.25, TPSA 63.60 Å^2^, log *P* 2.66) and full compliance with Lipinski's rule of five. Although compound 1 showed high Caco-2 permeability, its predicted oral bioavailability remained low (*F*_50%_ low), suggesting that its therapeutic effect may be primarily localized within the gastrointestinal tract.^33,34^ In contrast, compound 6 demonstrated superior pharmacokinetic performance with high predicted oral bioavailability (*F*_50%_), likely due to its optimized cholestane scaffold and extended C-24 side chain which enhances hydrophobic interactions and membrane permeation.^35,36^ This high systemic exposure potential, coupled with its potent α-glucosidase inhibition (IC_50_ = 2.0 µM), identifies 6 as a highly promising candidate for further development.

Structure–property relationship analysis revealed that structural modifications significantly impact both bioactivity and pharmacokinetics. The 3β-hydroxyl group in 2 and 6 facilitates dual hydrogen-bond interactions, whereas oxidation to a 3-ketone in 3 or benzoylation in 7 reduces potency and alters permeability due to loss of H-bond donors or increased steric hindrance.^37^ Similarly, excessive glycosylation in 5 (TPSA > 150 Å^2^) led to a dramatic decline in both α-glucosidase inhibition and Caco-2 permeability, confirming that bulky, polar sugar moieties hinder passive diffusion and optimal active site fit.^33^ Regarding distribution, the high plasma protein binding (PPB > 98%) and low unbound fractions (*f*_u_ < 3%) of 6 and 7 suggest a potential for localized gastrointestinal activity, which is therapeutically relevant for α-glucosidase inhibitors.^38–40^ Safety assessments indicated low risks for hERG blockade and nephrotoxicity across the series.^41^ However, the predicted DILI and endocrine-related interactions (ER/AR) for 1 and 6 represent preliminary *in silico* alerts that warrant further *in vivo* validation.^42^ Overall, the integration of ADMET and potency data identifies 1 as a balanced lead for structural optimization and 6 as a potent candidate with high systemic exposure potential, together providing a robust foundation for developing steroid-based antidiabetic agents.

## Experimental

### General experimental procedures

UV-vis spectrum was recorded using a spectrophotometer (Shimadzu Pte., Ltd, Singapore) while CD and FTIR spectra were recorded using a Jasco CD spectrometer J-1100 and a JASCO FT/IR-6600 spectrometer, respectively. HRESIMS data were acquired on a Xevo G2-XS QTOF mass spectrometer (Waters, USA). 1D and 2D-NMR (500 MHz) data were obtained on a Bruker Avance III (Bruker BioSpin AG, Fällanden, Switzerland), with chemical shifts referenced to the residual solvent signals.

### Chemicals and reagents

Chromatographic separations employed silica gel 60, and Kieselgel 60F254 or RP-18F254 plates (Merck). α-Glucosidase (EC 3.2.1.20) from *Saccharomyces cerevisiae* and *p*-nitrophenyl-α-d-glucopyranoside were purchased from Sigma-Aldrich. Acarbose and DMSO were obtained from Merck (Darmstadt, Germany). All solvents and additional reagents used in this study were of analytical grade or the highest purity commercially available.

### Plant material

A 7.4 kg batch of dried *S. torvum* fruits was obtained from an initial 30 kg of fresh material collected in Lam Dong province (Vietnam) in December 2018. Authentication was conducted by Dr Anh Tuan Dang-Le, and the voucher specimen DMC-9003 was archived at the Faculty of Chemistry, VNUHCM-University of Science. The dehydration process involved washing the unripe fruits, followed by exposure to sunlight until fully dried.

### Preparation of extracts and isolation of purified compounds

Dried unripe fruits of *S. torvum* were defatted with *n*-hexane, then extracted with EtOAc to yield extract EE (90.0 g). EE was fractionated by NP-SiO_2_ CC (acetone-*n*-hexane, 0 : 100 → 90 : 10) to afford 22 fractions (EE1–EE22). Fraction EE8 (0.8 g) was separated by NP-SiO_2_ CC (CHCl_3_-*n*-hexane, 0–100% CHCl_3_) and monitored by TLC to give seven subfractions (EE8.1–EE8.7). Subfr. EE8.5 (176.8 mg) was purified by NP-SiO_2_ CC (EtOAc–*n*-hexane, 0–100% EtOAc), followed by preparative TLC using an EtOAc–*n*-hexane system (30 : 70) to yield 6 (3.1 mg). Subfr. EE8.6 (133.4 mg) was subjected to NP-SiO_2_ CC (EtOAc–*n*-hexane, 0–100% EtOAc), followed by preparative TLC (*i*-PrOH–*n*-hexane, 5 : 95) to afford 1 (2.4 mg). Subfr. EE8.7 (402.9 mg) was chromatographed on NP-SiO_2_ (EtOAc–*n*-hexane, 0–100% EtOAc) to give three subfractions, including EE8.7.1–EE8.7.3. Subfr. EE8.7.2 (57.1 mg) was washed with MeOH–*n*-hexane and recrystallized from CHCl_3_ to give 3 (25.0 mg). Subfr. EE8.7.3 (80.8 mg) was further purified by NP-SiO_2_ CC (*i*-PrOH–*n*-hexane, 0–100% *i*-PrOH) to yield 7 (37.3 mg). Fraction EE12 (0.8 g) was separated by NP-SiO_2_ CC (acetone–*n*-hexane, 0–100% acetone) into three subfractions (EE12.1–EE12.3). Subfraction EE12.1 (35.3 mg) was purified by RP-SiO_2_ CC (MeCN–H_2_O, 0–100% MeCN) to yield 5 (3.5 mg). Subfraction EE12.2 (89.5 mg) was chromatographed on NP-SiO_2_ CC (acetone–*n*-hexane, 0–100% acetone), then preparative RP-TLC (MeCN–MeOH–H_2_O, 4 : 5 : 1) to give 2 (4.7 mg). Subfraction EE12.3 (94.0 mg) was purified by RP-SiO_2_ CC (MeCN–H_2_O, 0–100% MeCN) to afford 4 (10.0 mg).

Solacanin C (1): white amorphous powder; ^1^H (500 MHz, CDCl_3_) and ^13^C NMR (125 MHz, CDCl_3_), see Table 1; [*α*]^25^_D_ = +14.4 (*c* = 0.03 g/100 mL, MeOH); IR *ν*_max_ 3399 cm^−1^, 1729 cm^−1^, 1665 cm^−1^, 1029 cm^−1^; HRESIMS *m*/*z* 375.2528 [M + H]^+^ (C_23_H_35_O_4_^+^, 375.2530).

Neochlorogenin (2): ^1^H NMR (500 MHz, CDCl_3_): *δ*_H_ (*J* in Hz) 4.41 (1H, q, *J* = 7.5 Hz), 3.95 (1H, dd, *J* = 11.0, 2.8 Hz), 3.57 (1H, *m*), 3.43 (1H, ddd, *J* = 10.7, 10.7, 4.5 Hz), 3.29 (1H, br d, *J* = 11.0 Hz), 1.07 (3H, d, *J* = 7.1 Hz), 0.97 (3H, d, *J* = 7.1 Hz), 0.83 (3H, s), 0.76 (3H, s), 0.69 (1H, td, *J* = 12.4, 2.5 Hz). ^13^C NMR (125 MHz, CDCl_3_): *δ*_C_ (ppm) 109.7, 80.8, 71.2, 69.4, 65.2, 62.0, 56.0, 53.8, 51.7, 42.2, 41.8, 39.9, 37.2, 36.4, 33.9, 32.2, 31.7, 31.0, 29.7, 27.1, 25.9, 25.8, 21.0, 16.4, 16.0, 14.3, 13.5. IR *ν*_max_ 3309 cm^−1^, 1041 cm^−1^; HRESIMS *m*/*z* 455.3158 [M + Na]^+^ (C_27_H_44_NaO_4_^+^, 455.3137).

Neoscorpiogenin (3): ^1^H NMR (500 MHz, CDCl_3_): *δ*_H_ (*J* in Hz) 4.41 (1H, q, *J* = 7.5 Hz), 3.93 (1H, dd, *J* = 11.0, 2.8 Hz), 3.48 (1H, ddd, *J* = 10.8, 10.8, 4.5 Hz), 3.28 (1H, br d, *J* = 11.0 Hz), 2.71 (1H, dd, *J* = 15.8, 3.1 Hz), 2.37 (1H, td, *J* = 15.5, 6.7 Hz), 2.30 (1H, m), 2.19 (1H, dd, *J* = 15.8, 14.5 Hz), 1.07 (3H, d, *J* = 7.1 Hz), 1.02 (3H, s), 0.99 (3H, d, *J* = 6.7 Hz), 0.78 (3H, s). ^13^C NMR (125 MHz, CDCl_3_): *δ*_C_ (ppm) 211.6, 109.9, 80.9, 70.0, 69.9, 65.3, 62.1, 55.9, 53.5, 53.2, 42.3, 41.9, 39.9, 39.6, 38.6, 37.9, 34.0, 31.9, 29.8, 27.2, 26.1, 25.9, 21.3, 16.6, 16.2, 14.4, 12.9. IR *ν*_max_ 3423 cm^−1^, 1714 cm^−1^, 1046 cm^−1^; HRESIMS *m*/*z* 431.3180 [M + H]^+^ (C_27_H_43_O_4_^+^, 431.3161).

Neochlorogenin 6-O-β-d-quinovopyranoside (4): ^1^H NMR (500 MHz, CDCl_3_): *δ*_H_ (*J* in Hz) 4.44 (1H, ddd, *J* = 14.8, 7.1 Hz), 4.27 (1H, d, *J* = 7.3 Hz), 3.96 (1H, d, *J* = 10.4 Hz), 3.55 (1H, m), 3.48 (1H, m), 3.37 (1H, m), 3.33 (1H, ddd, *J* = 10.4, 7.1 Hz), 3.22 (1H, m), 3.15 (1H, dd, *J* = 9.1, 9.0 Hz), 3.15 (1H, dd, *J* = 9.0 Hz), 1.30 (3H, d, *J* = 4.8 Hz), 1.09 (3H, d, *J* = 6.9 Hz), 1.00 (3H, d, *J* = 6.9 Hz), 0.87 (3H, s), 0.79 (3H, s). ^13^C NMR (125 MHz, CDCl_3_): *δ*_C_ (ppm) 109.7, 104.3, 80.8, 80.3, 76.4, 75.4, 74.2, 71.6, 70.9, 65.1, 62.1, 56.2, 53.6, 50.4, 42.2, 40.6, 40.6, 39.8, 37.3, 36.5, 34.0, 31.9, 31.4, 30.2, 27.1, 25.8, 22.7, 21.0, 17.8, 16.5, 16.1, 14.3, 13.6. IR *ν*_max_ 3393 cm^−1^, 1069 cm^−1^; HRESIMS *m*/*z* 579.3891 [M + H]^+^ (C_33_H_55_O_8_^+^, 579.3897).

Neochlorogenin 6-O-α-l-rhamnopyranosyl-(1 → 3)-β-d-quinovopyranoside (5): ^1^H NMR (500 MHz, CD_3_OD): *δ*_H_ (*J* in Hz) 5.10 (1H, d, *J* = 1.8 Hz), 4.40 (1H, ddd, *J* = 9.7, 7.8, 7.7 Hz), 4.27 (1H, d, *J* = 7.9 Hz), 3.99 (1H, dddd, *J* = 9.5, 9.6, 3.4, 3.4 Hz), 3.94 (1H, dd, *J* = 5.3, 2.0 Hz), 3.70 (1H, dd, *J* = 9.5, 3.4 Hz), 3.44 (1H, m), 3.41 (1H, m), 3.38 (1H, m), 3.37 (1H, m), 3.33 (1H, ddd, *J* = 10.5, 3.0, 2.9 Hz), 3.29 (1H, m), 3.29 (1H, m), 3.27 (1H, m), 3.02 (1H, t, *J* = 9.2 Hz), 1.27 (3H, d, *J* = 7.3 Hz), 1.24 (3H, d, *J* = 6.2 Hz), 1.08 (3H, d, *J* = 7.1 Hz), 0.99 (3H, d, *J* = 6.9 Hz), 0.87 (3H, s), 0.79 (3H, s). ^13^C NMR (125 MHz, CD_3_OD): *δ*_C_ (ppm) 111.1, 105.1, 102.8, 84.5, 82.3, 80.5, 76.4, 75.8, 74.0, 73.0, 72.4, 72.3, 71.9, 70.1, 66.1, 63.6, 57.4, 55.1, 51.8, 43.5, 41.7, 41.6, 41.0, 37.6, 35.2, 33.0, 32.7, 32.7, 31.9, 28.5, 27.0, 26.7, 22.1, 18.3, 17.9, 16.9, 16.4, 14.7, 13.8. IR *ν*_max_ 3382 cm^−1^, 1070 cm^−1^; HRESIMS *m*/*z* 725.4486 [M + H]^+^ (C_39_H_65_O_12_^+^, 725.4471).

Debenzoylcarpesterol (6): ^1^H NMR (500 MHz, CDCl_3_): *δ*_H_ (*J* in Hz) 5.68 (1H, s), 3.74 (1H, d, *J* = 9.6 Hz), 3.18 (1H, m), 1.16 (3H, d, *J* = 6.0 Hz), 0.94 (3H, d, *J* = 6.8 Hz), 0.89 (3H, d, *J* = 6.6 Hz), 0.89 (3H, d, *J* = 6.6 Hz), 0.86 (3H, s), 0.81 (3H, d, *J* = 6.9 Hz), 0.60 (3H, s). ^13^C NMR (125 MHz, CDCl_3_): *δ*_C_ (ppm) 200.7, 160.8, 124.0, 76.6, 71.3, 60.3, 55.1, 53.3, 51.4, 45.2, 42.8, 41.7, 39.1, 36.8, 35.0, 30.4, 29.9, 29.8, 29.1, 27.2, 23.8, 22.7, 21.9, 20.6, 17.9, 17.6, 14.8, 12.7, 12.4, 11.9. IR *ν*_max_ 3421 cm^−1^, 1716 cm^−1^, 1670 cm^−1^, 1023 cm^−1^; HRESIMS *m*/*z* 459.3833 [M + H]^+^ (C_30_H_51_O_3_^+^, 459.3833).

Carpesterol (7): ^1^H NMR (500 MHz, CDCl_3_): *δ*_H_ (*J* in Hz) 8.06 (2H, d, *J* = 7.7 Hz), 7.56 (1H, dd, *J* = 7.7, 7.6 Hz), 7.44 (2H, dd, *J* = 7.7, 7.6 Hz), 5.69 (1H, s), 4.69 (1H, ddd, *J* = 10.3, 10.3, 4.3 Hz), 3.73 (1H, br d, *J* = 10.6 Hz), 2.27 (1H, d, *J* = 10.6 Hz), Hz), 2.21 (1H, dd, *J* = 5.5 Hz), 1.10 (3H, d, *J* = 5.5 Hz), 1.03 (1H, dd, *J* = 14.3, 9.9 Hz), 0.95 (3H, d, *J* = 6.8 Hz), 0.92 (3H, s), 0.91 (3H, d, *J* = 6.7 Hz), 0.89 (3H, m), 0.81 (3H, *d*, *J* = 6.8 Hz), 0.62 (3H, s). ^13^C NMR (125 MHz, CDCl_3_): *δ*_C_ (ppm) 200.3, 166.6, 161.1, 133.0, 130.7, 129.7, 128.5, 123.8, 79.1, 71.2, 60.2, 55.1, 53.3, 51.2, 45.2, 42.8, 41.6, 39.5, 39.0, 36.4, 31.9, 30.2, 29.0, 27.2, 26.3, 23.8, 22.7, 21.9, 20.6, 17.8, 17.6, 14.8, 12.6, 12.5, 12.0. IR *ν*_max_ 3503 cm^−1^, 1713 cm^−1^, 1675 cm^−1^, 1275 cm^−1^; HRESIMS *m*/*z* 563.4096 [M + H]^+^ (C_37_H_55_O_4_^+^, 563.4095).

### Assay for α-glucosidase inhibitory effect

The α-glucosidase inhibitory activity was operated using a modified *p*-nitrophenyl-α-d-glucopyranoside (*p*-NPG) spectrophotometric assay adapted to our laboratory conditions. Briefly, 50 µL *p*-NPG (1.5 mM) and 50 µL α-glucosidase (0.1 U mL^−1^) in 0.01 M phosphate buffer pH 7.0 were added to 525 µL of test sample to obtain a final volume of 625 µL. The reaction was incubated at 37 °C for 30 min and terminated by adding 375 µL of 0.1 M Na_2_CO_3_. The released *p*-nitrophenol was converted to *p*-nitrophenolate and quantified at 401 nm. Acarbose was used as the positive control. Inhibitory activity was expressed as percentage inhibition (*I*%) and IC_50_ values.^43^ All experiments were performed in triplicate (*n* = 3). Data were analyzed by one-way ANOVA in Microsoft Excel 365 and are presented as mean ± SD, with statistical significance set at *p* < 0.05.

### Molecular docking simulation

The amino acid sequence of MAL12 from *Saccharomyces cerevisiae* (strain S288C; α-glucosidase) was obtained from UniProt P53341 and generated using ColabFold v1.5.5: AlphaFold2 using MMseqs2 with default settings. The best-performing relaxed model was then selected for further use. All-atom refinement and structural validation were carried out using MolProbity. The geometry assessment from MolProbity (Table S2) fell within the range expected for a homology model overall.^44,45^ Three residues including Thr2, Ile3, and Ala278 were flagged as Ramachandran outliers. However, these outliers reflect minor backbone dihedral deviations rather than substantial sidechain distortion, and none of these residues corresponds to a catalytic triad residue. Therefore, these residues were unlikely to have a major impact on the binding poses in docking simulation. The resulting MolProbity-optimized coordinates served as input for subsequent molecular docking studies. The 3D conformation of compound 1–7 was generated based on SMILES format by OpenBabel tool. The positive control acarbose was also docked with homology model of α-glucosidase. All compounds and protein structure were prepared before performing docking by removing non-standard residues, adding missing hydrogen atoms using the Dock Prep tool on Chimera v1.17.3.^46^ Autodock Vina version 1.1.2 was used to perform the molecular docking interaction in this study.^47^ Molecular docking was performed using a grid box centered at coordinates (1.25, 3.85, −2.13) Å, with corresponding dimensions of 24.31 Å × 25.43 Å × 20.76 Å. PyMOL 3.0 software was employed to visualize 3D docking poses, while Biovia Discovery Studio 21.1 was used to illustrate 2D interactions between the enzyme and the compounds.

## Conclusions

In this study, seven steroidal compounds from the unripe fruits of *S. torvum* successfully isolated and identified, including the novel pregnane steroid solacanin C (1) and six known compounds (2–7). A biological evaluation demonstrated that all tested steroids possess superior α-glucosidase inhibitory activity compared to the standard acarbose. The integration of *in vitro* assays and *in silico* molecular docking provided a clear rationale for their inhibitory mechanisms, highlighting the importance of the steroid core's orientation and specific functional groups at the C-3 and C-6 positions for enzyme binding. ADMET profiling revealed that 6 exhibited superior oral bioavailability (*F*_50%_) and high Caco-2 permeability (log *P*_app_ > −5.15), indicating excellent systemic exposure. A new compound 1 demonstrated the most balanced medicinal chemistry profile, with the highest QED score and full compliance with drug-likeness filters. These results, combined with low predicted toxicity risks, identify 1 as the most tractable lead and 6 as a highly promising candidate for further development.

## Author contributions

Tho Huu Le: conceptualization (lead), computational studies (lead), bioassay (equal), methodology (equal), project administration (equal), writing – original draft preparation (lead), writing – review and editing (equal); Nguyen Thien Han Le: bioassay (equal), data curation (equal); Phu Hoang Dang: data curation (equal), writing – original draft preparation (supporting); Tung Thanh Phan: project administration (equal), writing – original draft preparation (supporting), conceptualization (supporting), Phong Thanh Nguyen: conceptualization (supporting), bioassay (equal), Hai Xuan Nguyen, Truong Nhat Van Do: data curation (equal), bioassay (supporting); Mai Thanh Thi Nguyen: supervision (lead), writing – review and editing (equal). All authors have read and agreed to the published version of the manuscript.

## Conflicts of interest

No potential conflict of interest was reported by the authors.

## Supplementary Material

RA-OLF-D6RA03305A-s001

## Data Availability

No data was used for the research described in this article. Supplementary information (SI) is available. See DOI: https://doi.org/10.1039/d6ra03305a.
