# Supplementary material for: Solacanin C, a new pregnane steroid from Solanum torvum fruits: structural elucidation, potent α-glucosidase inhibition, and in silico ADMET profiling
Source: RSC Adv. 2026 Aug 24;16(41):49389–97. doi: 10.1039/d6ra03305a (PMC13501368; doi:10.1039/d6ra03305a)
Supplement: Supplementary file 1 [file RA-016-D6RA03305A-s001.pdf]

Supplementary Information (SI) for RSC Advances.
This journal is © The Royal Society of Chemistry 2026

SUPPORTING INFORMATION

Solacanin C, a New Pregnane Steroid from *Solanum torvum* Fruits: Structural Elucidation, Potent α-Glucosidase Inhibition, and In Silico ADMET Profiling

Tho Huu Le,[a,b,c] Nguyen Thien Han Le,[a,b] Phu Hoang Dang,[a,b,c] Tung Thanh Phan,[a,b] Phong Thanh Nguyen,[a,b] Hai Xuan Nguyen,[a,b,c] Truong Nhat Van Do,[a,b,c] Mai Thanh Thi Nguyen[a,b,c,*]

[a]Faculty of Chemistry, University of Science, Ho Chi Minh City, 227 Nguyen Van Cu Street, Cho Quan Ward, Ho Chi Minh City 70000, Vietnam
[b]Vietnam National University Ho Chi Minh City, Linh Xuan Ward, Ho Chi Minh City 70000, Vietnam
[c]Research Lab for Drug Discovery and Development, University of Science, Ho Chi Minh City, Vietnam

*Corresponding Author:
Prof. Mai Thanh Thi Nguyen
Chancellor, Vietnam National University Ho Chi Minh City (VNUHCM)
E-mail: nttmai@hcmus.edu.vn
Tel: +84-907-426-331

Table S1. The α-glucosidase inhibitory activities result from all compounds isolated from unripe *Solanum torvum* fruits

**Compound**	**Inhibition percentage I (%)**					**IC_{50} (μM)**
	**250 μM**	**100 μM**	**50 μM**	**25 μM**	**10 μM**	
**5**	67.1 ± 1.1	36.8 ± 1.9	22.4 ± 2.0	8.4 ± 1.2	-	**145.5**
**7**	76.6 ± 3.1	48.5 ± 2.9	20.6 ± 0.6	9.5 ± 1.3	-	**110.1**
	**25 μM**	**10 μM**	**5 μM**	**2.5 μM**	**1 μM**	
**1**	92.0 ± 1.0	52.3 ± 1.5	31.9 ± 2.3	25.3 ± 1.1	15.8 ± 1.4	**9.4**
**2**	99.8 ± 2.3	96.6 ± 2.1	56.9 ± 1.0	36.1 ± 3.9	14.9 ± 3.8	**4.5**
**3**	91.8 ± 3.1	42.3 ± 3.6	21.8 ± 0.7	15.2 ± 4.0	-	**12.8**
**4**	96.2 ± 0.3	98.6 ± 0.6	90.7 ± 0.9	55.6 ± 2.6	2.5 ± 1.4	**2.3**
**6**	98.1 ± 0.5	82.4 ± 2.8	61.0 ± 4.5	26.6 ± 2.3	12.3 ± 3.1	**2.0**
**Acarbose[a]**						**190.6**

The inhibitory percentage (%) was calculated from the mean ± standard deviation (n = 3). The difference in inhibitory percentages between the concentrations in a test sample is statistically significant ($p < 0.05$).

–No inhibitory activity was shown

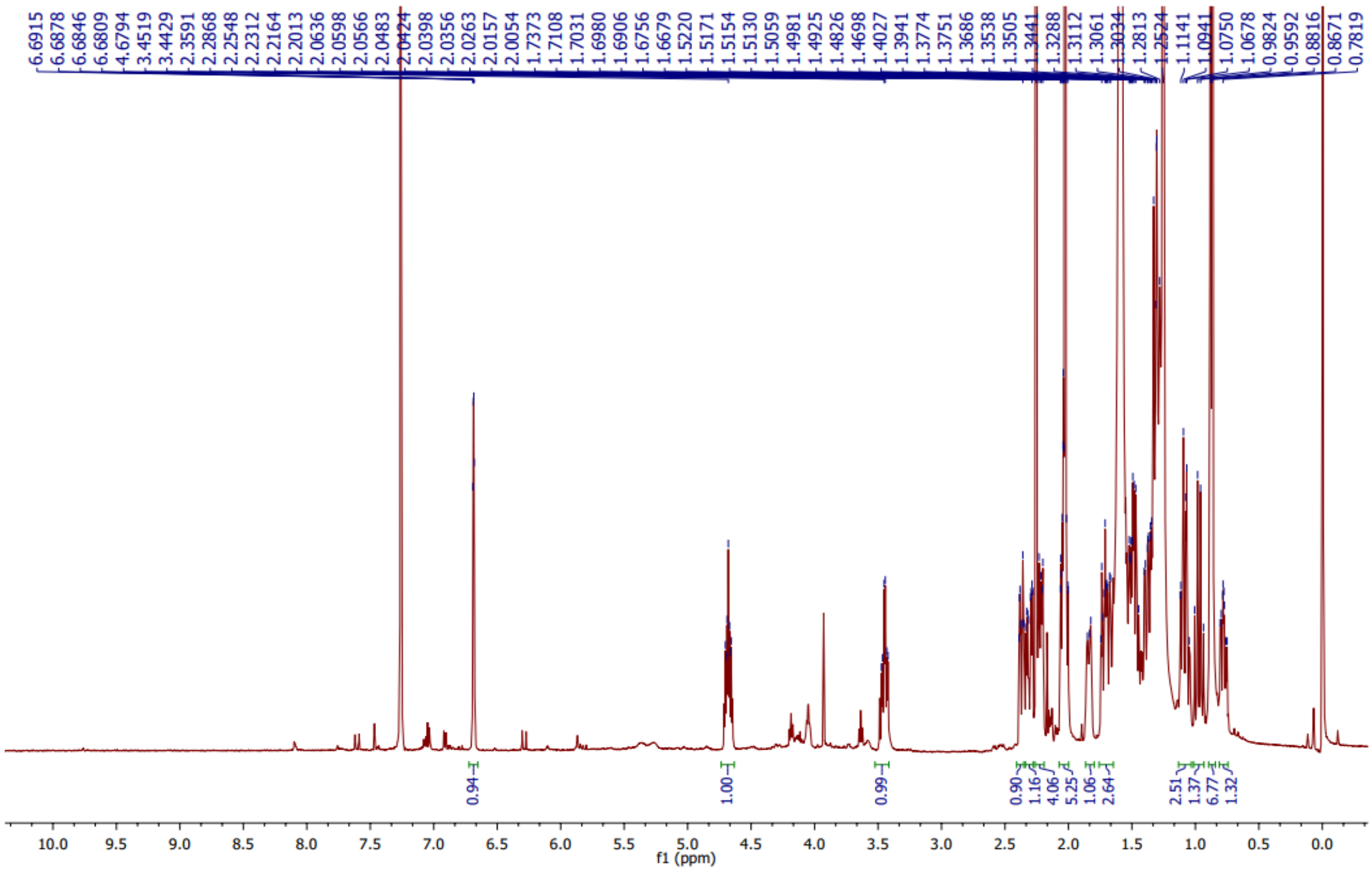

Figure S1. ^{1}H NMR spectrum of compound **1** (500 MHz, $CDCl_3$)

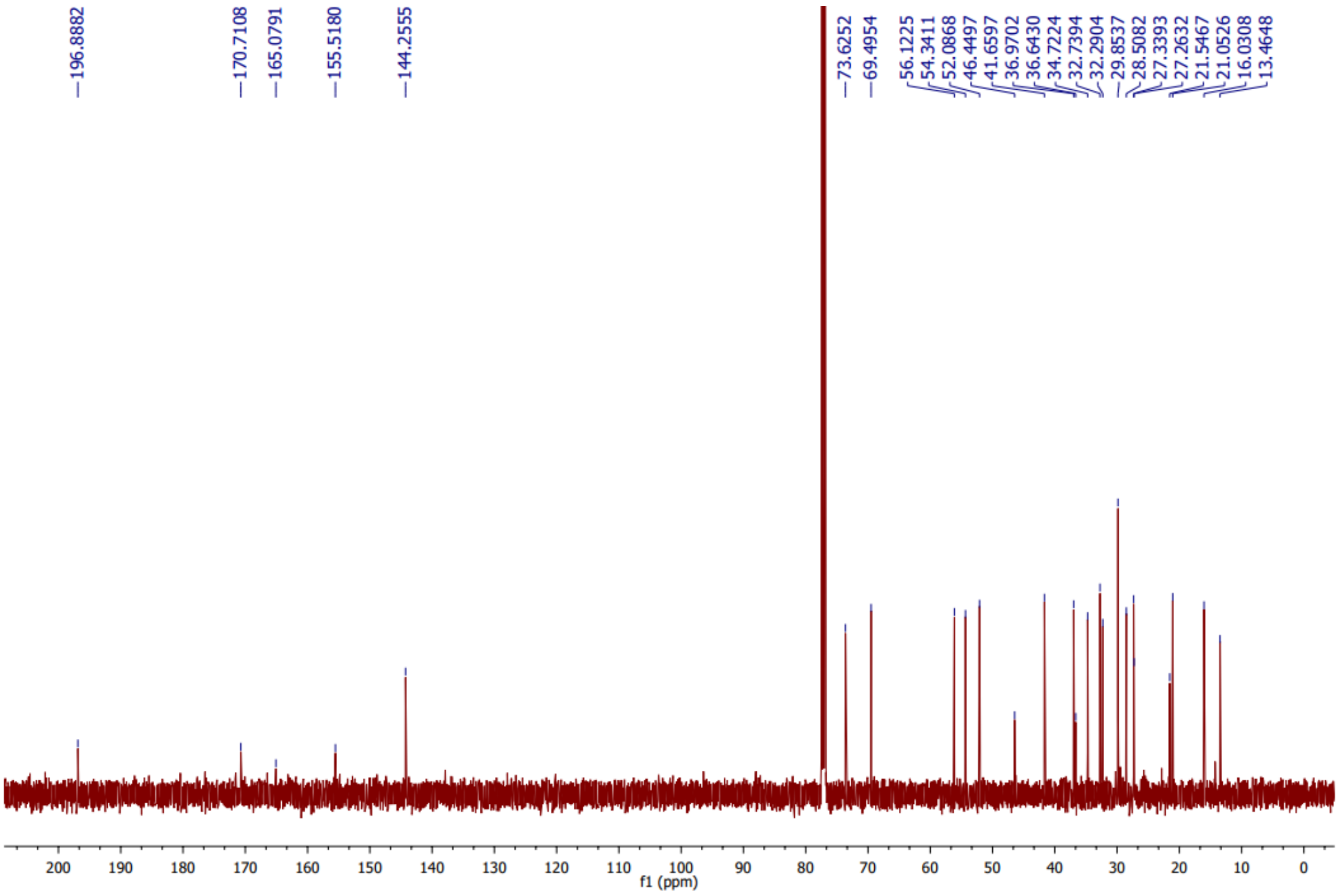

Figure S2. ^{13}C NMR spectrum of compound **1** (125 MHz, $CDCl_3$)

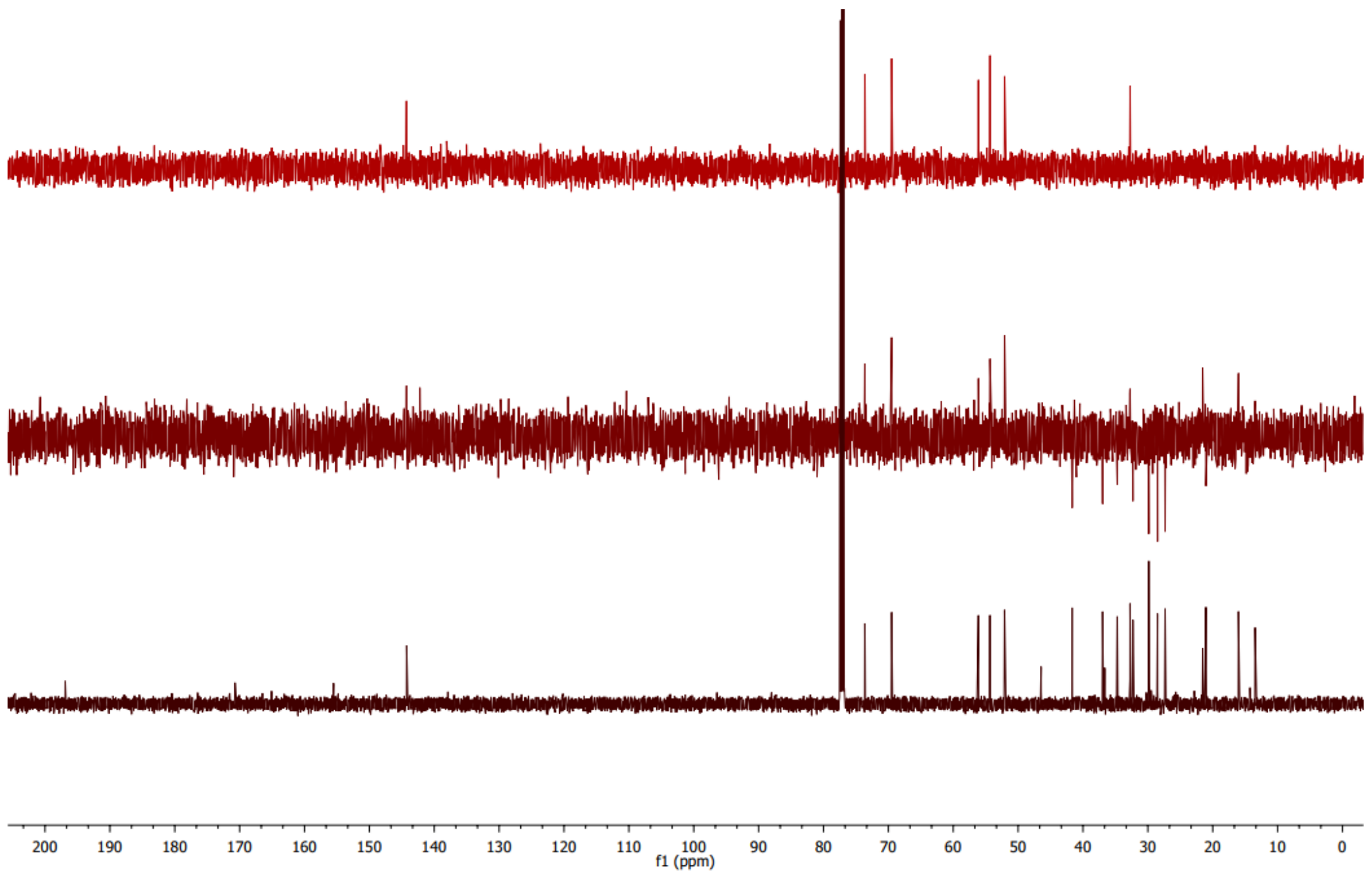

Figure S3. DEPT spectrum of compound **1** (125 MHz, $CDCl_3$)

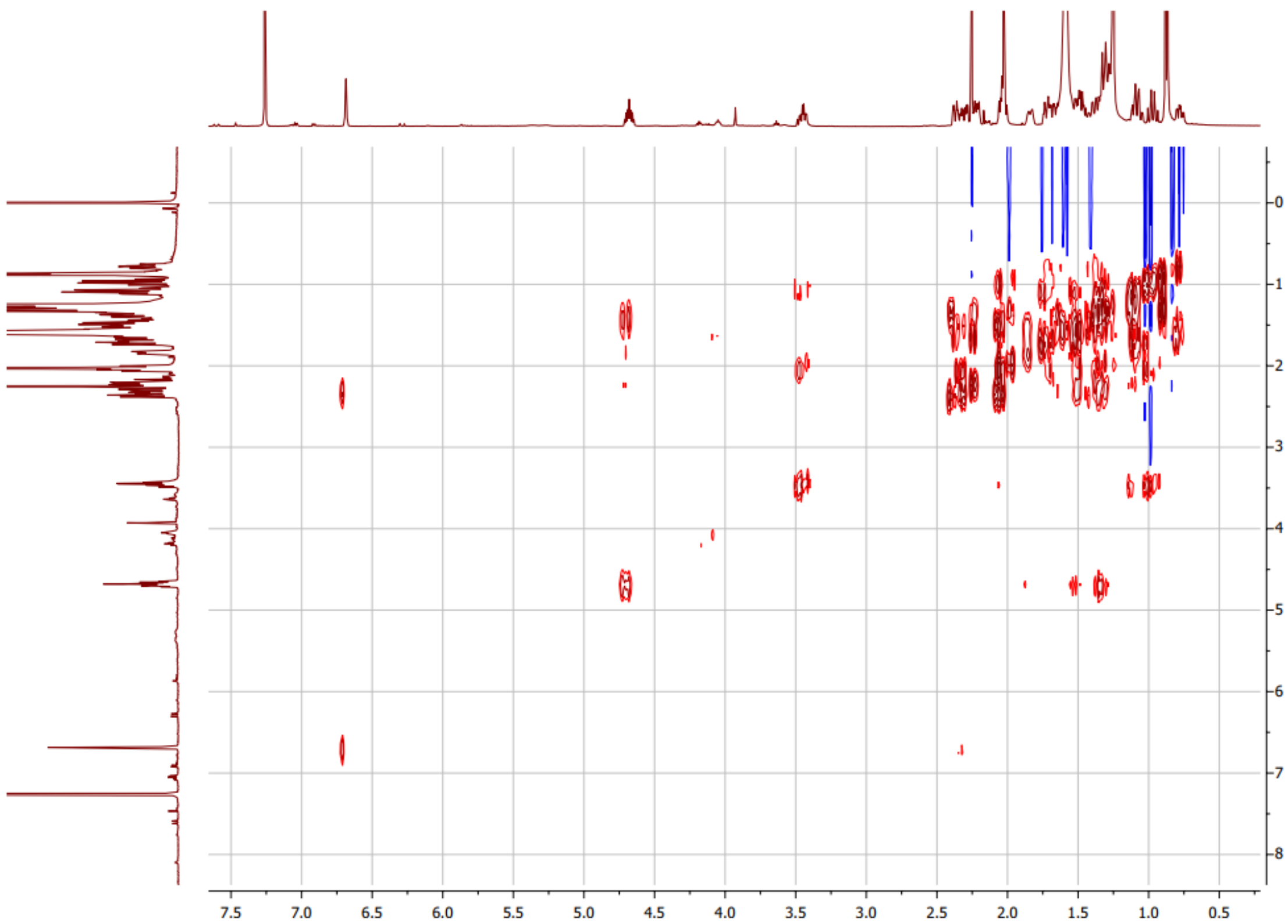

Figure S4. COSY spectrum of compound **1**

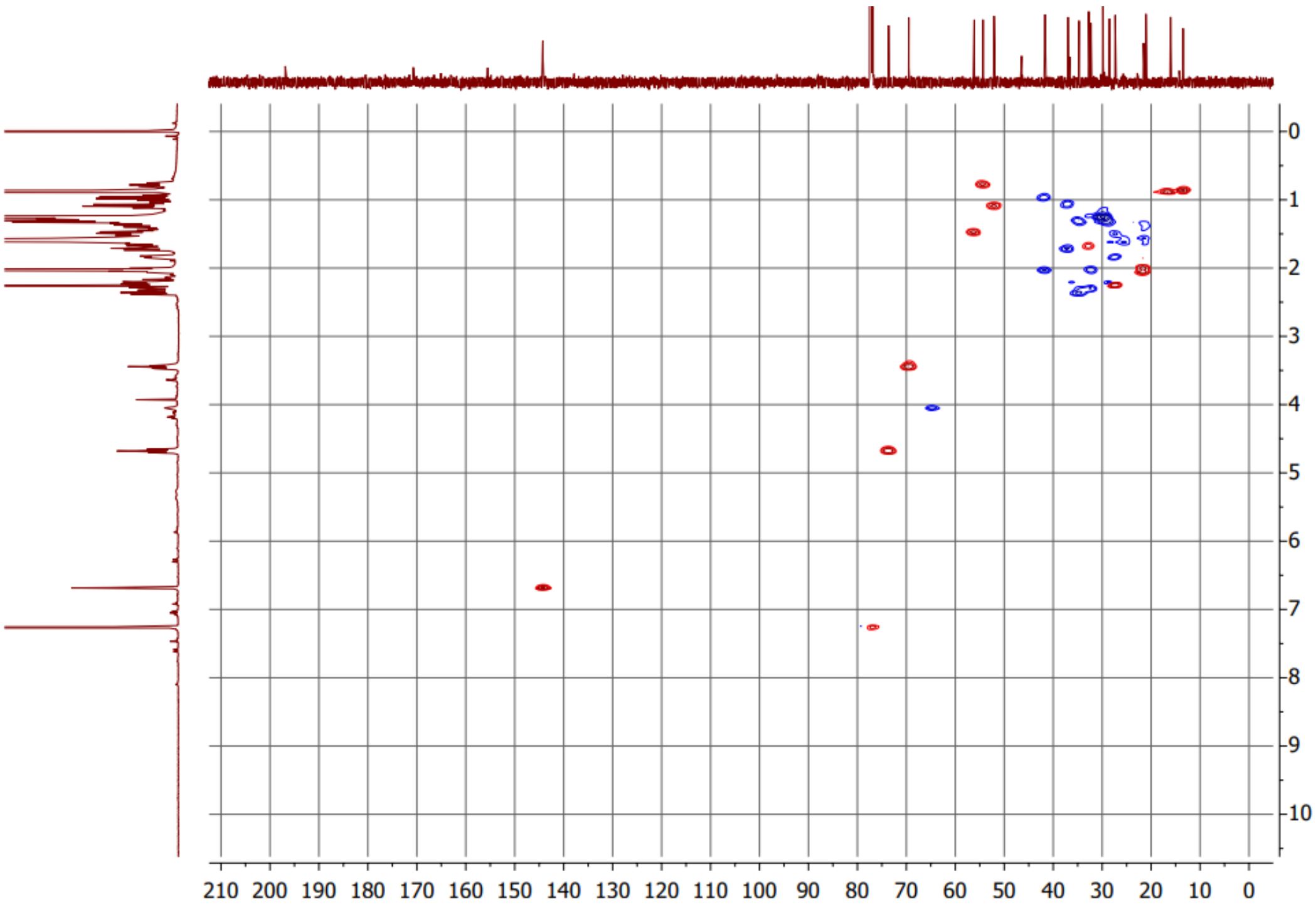

Figure S5. HSQC spectrum of compound **1**

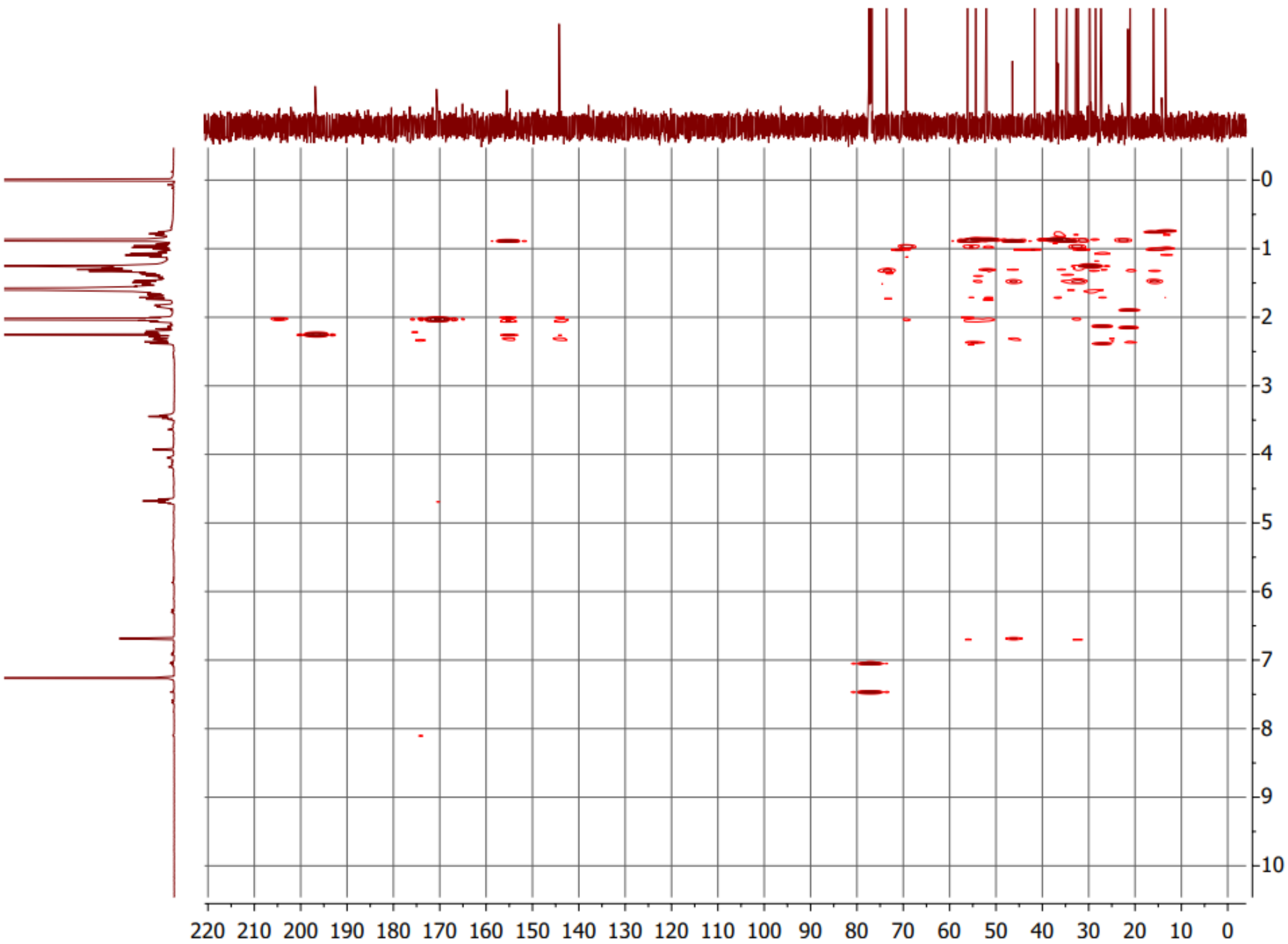

Figure S6. HMBC spectrum of compound **1**

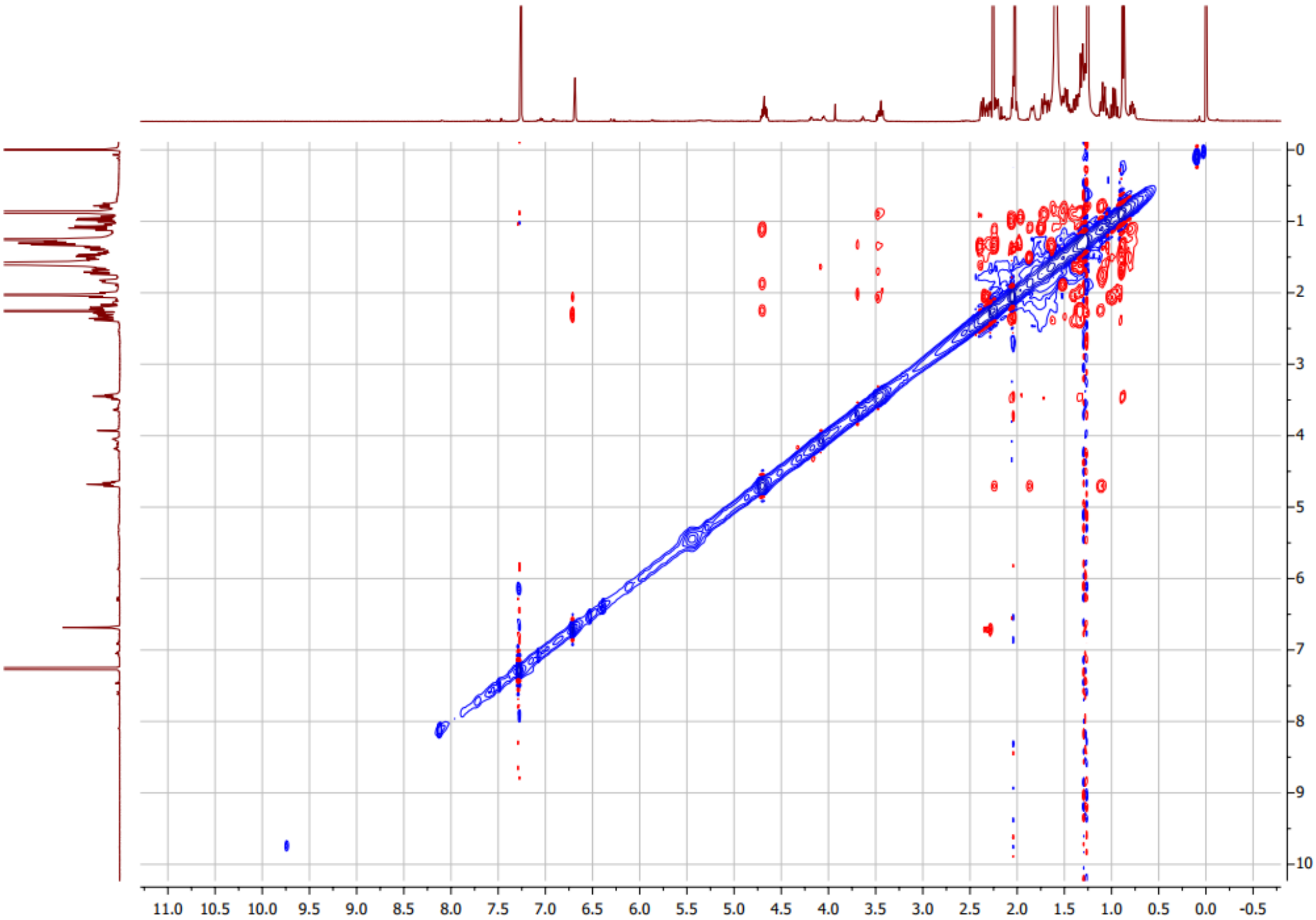

Figure S7. NOESY spectrum of compound **1**

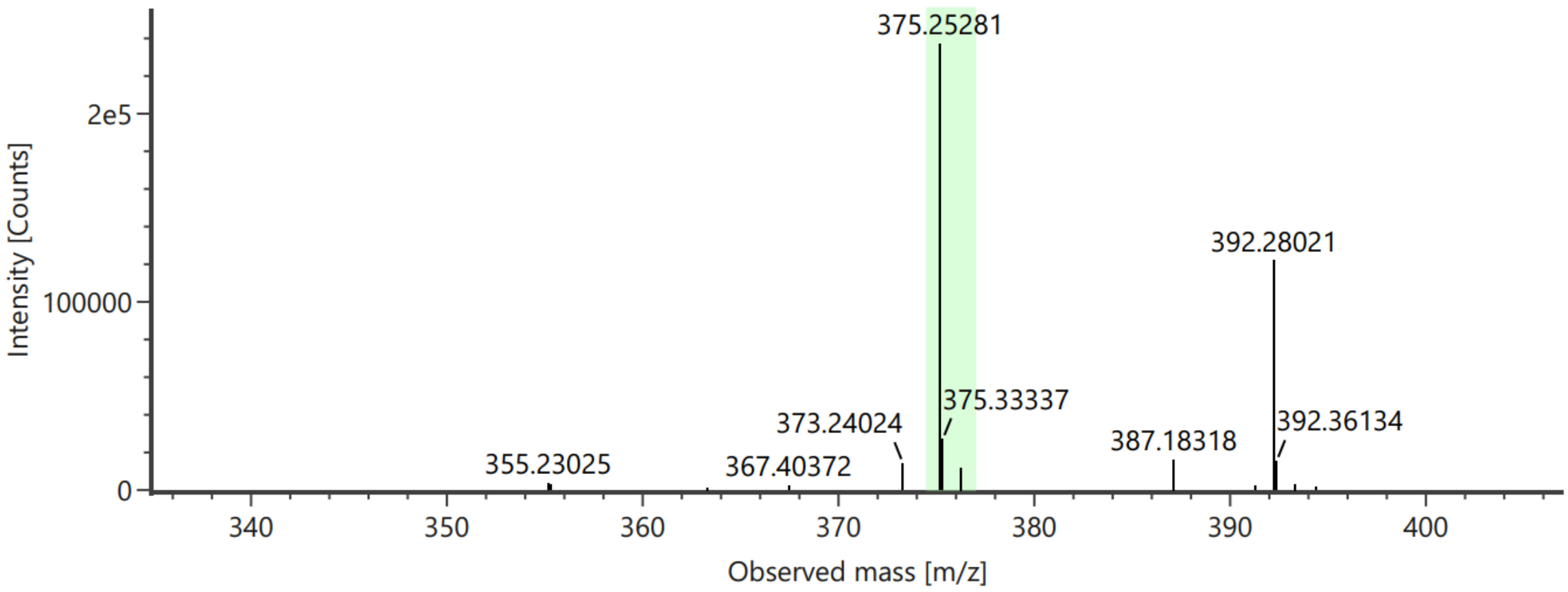

Figure S8. MS spectrum of compound **1**

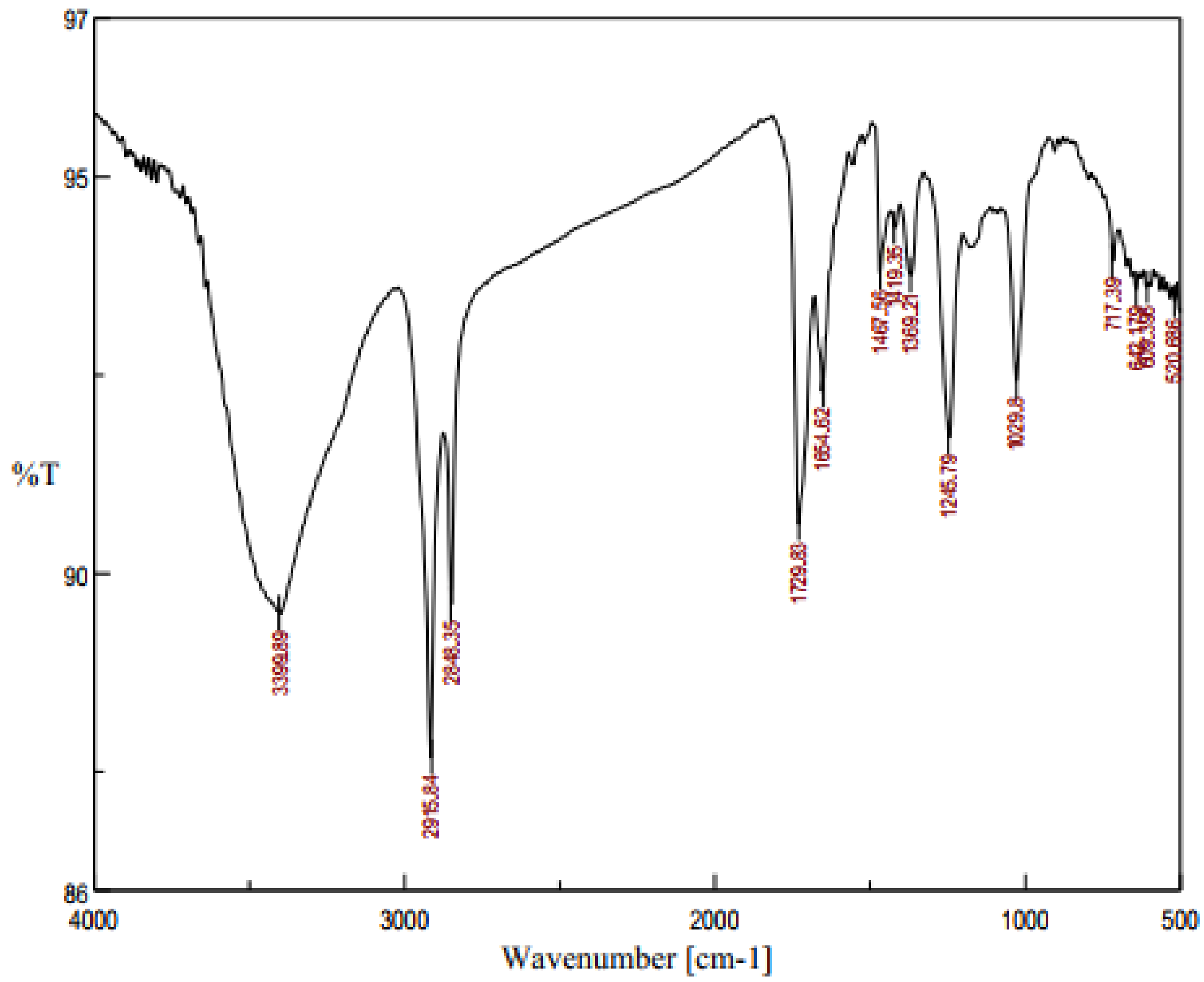

Figure S9. FTIR spectrum of compound **1**

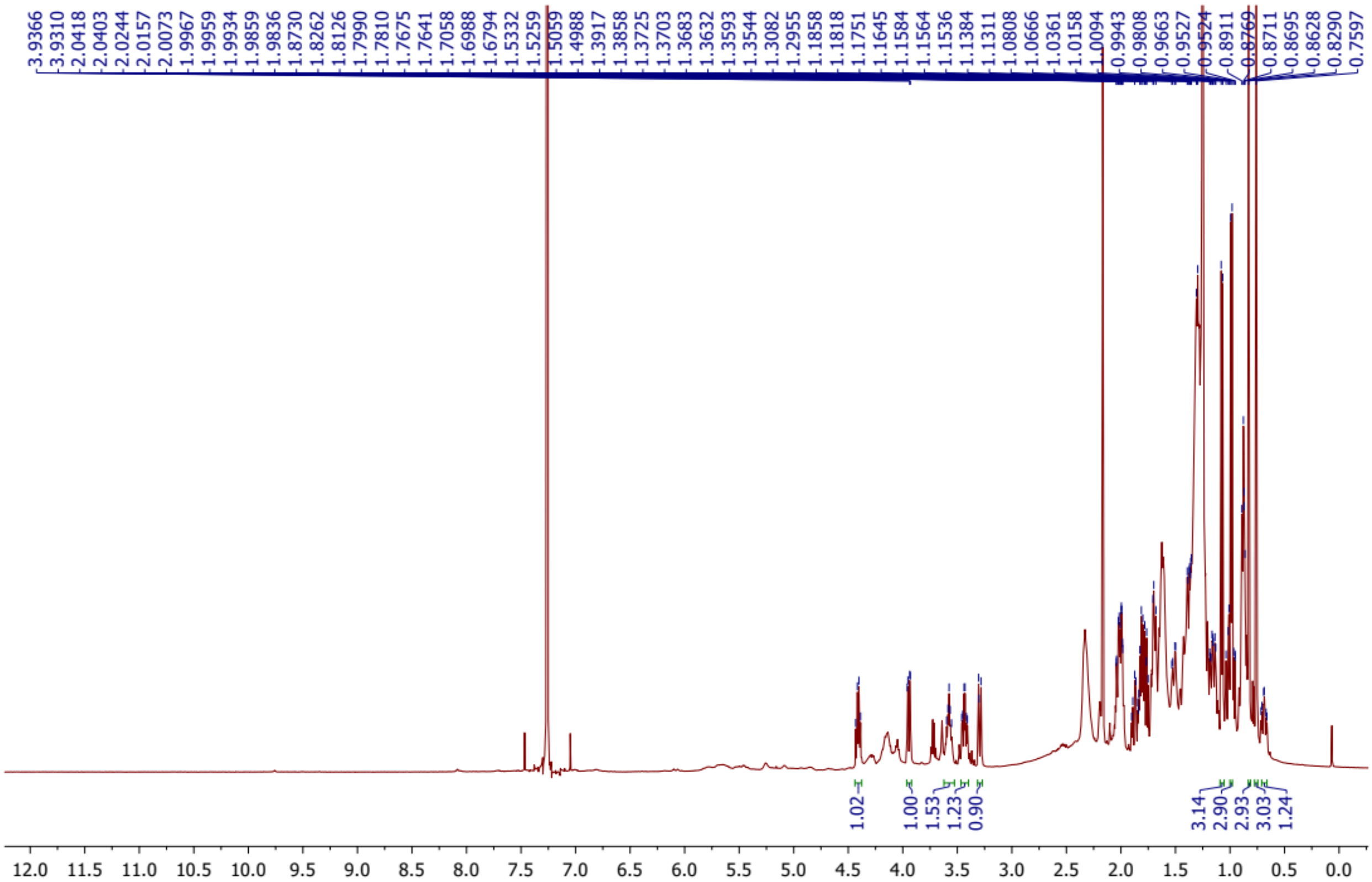

Figure S10. ^{1}H NMR spectrum of compound **2** (500 MHz, $CDCl_3$)

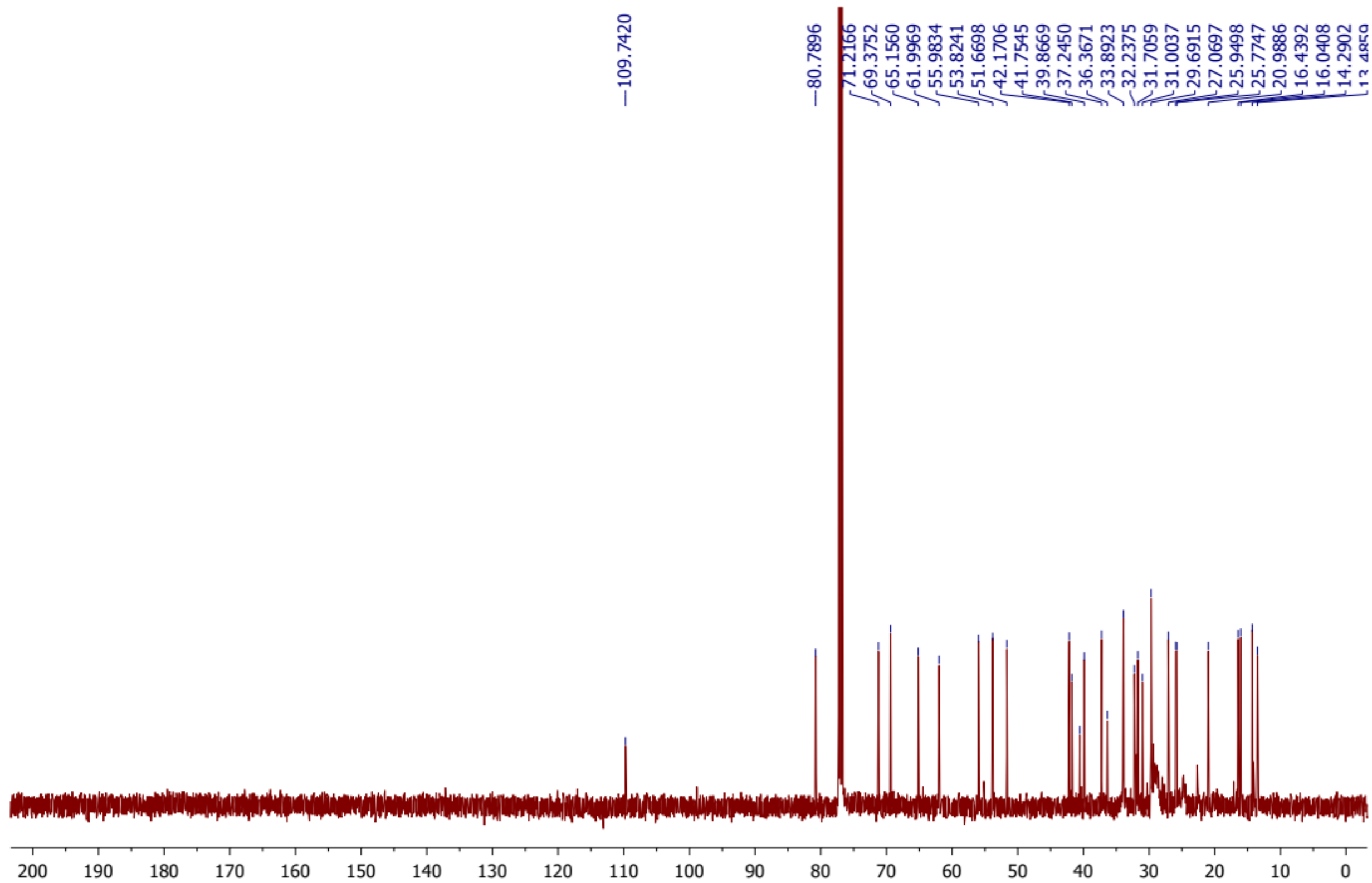

Figure S11. ^{13}C NMR spectrum of compound **2** (125 MHz, $CDCl_3$)

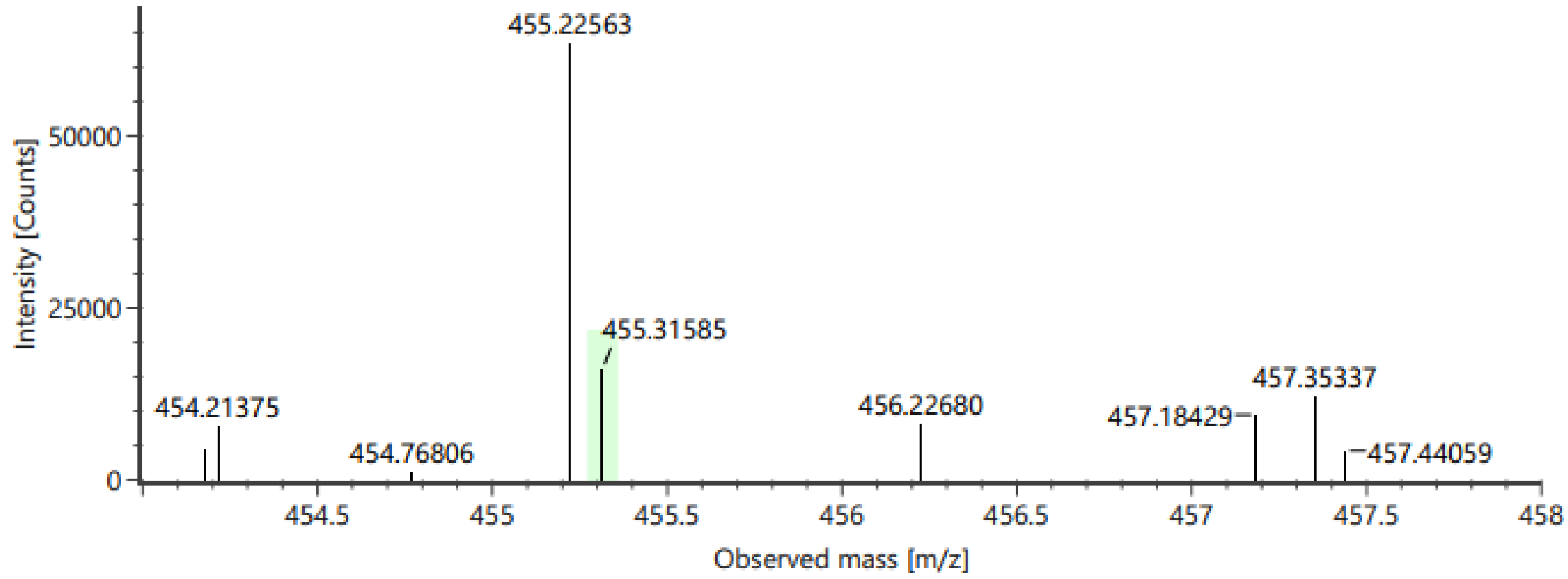

Figure S12. MS spectrum of compound **2**

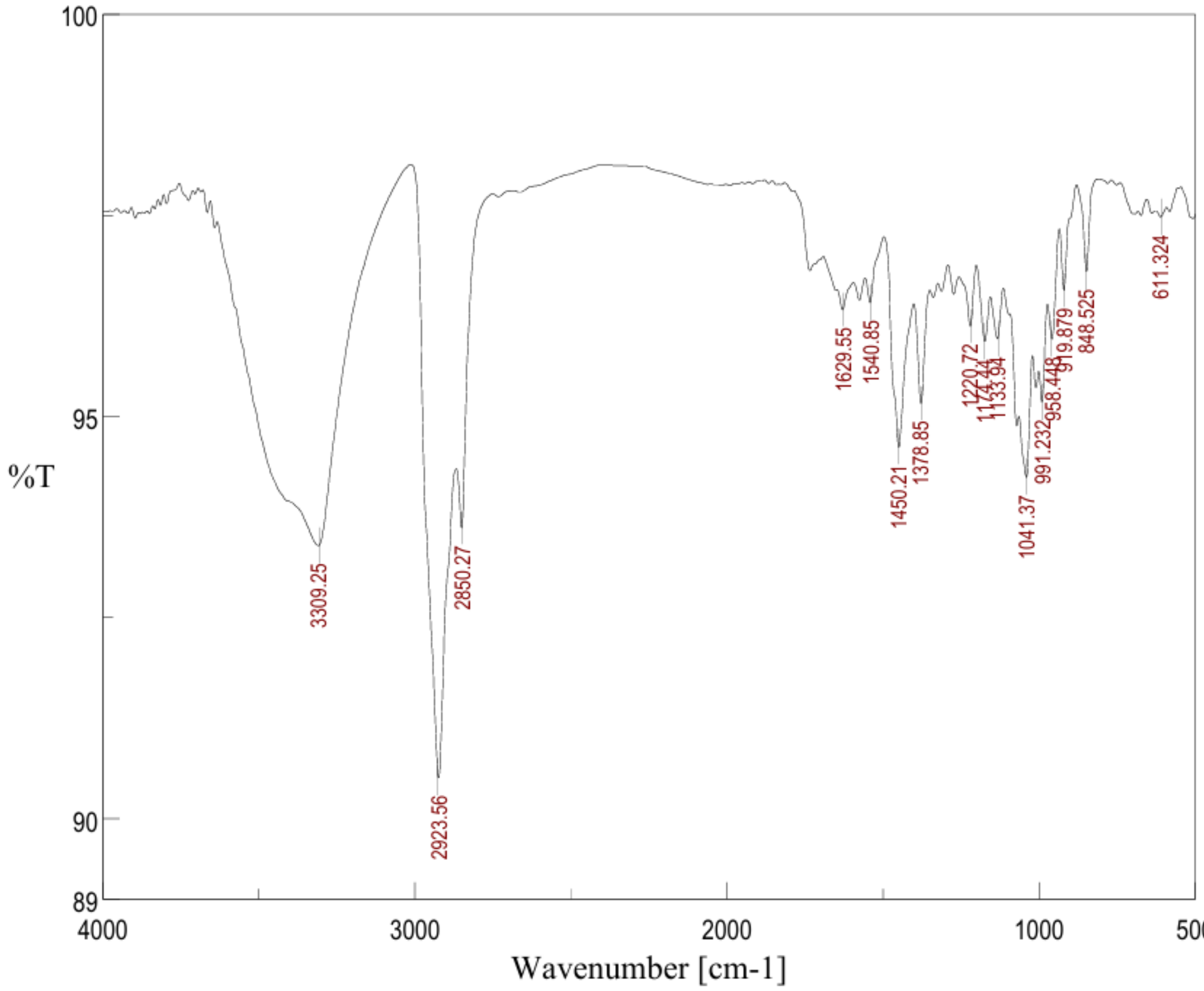

Figure S13. FTIR spectrum of compound **2**

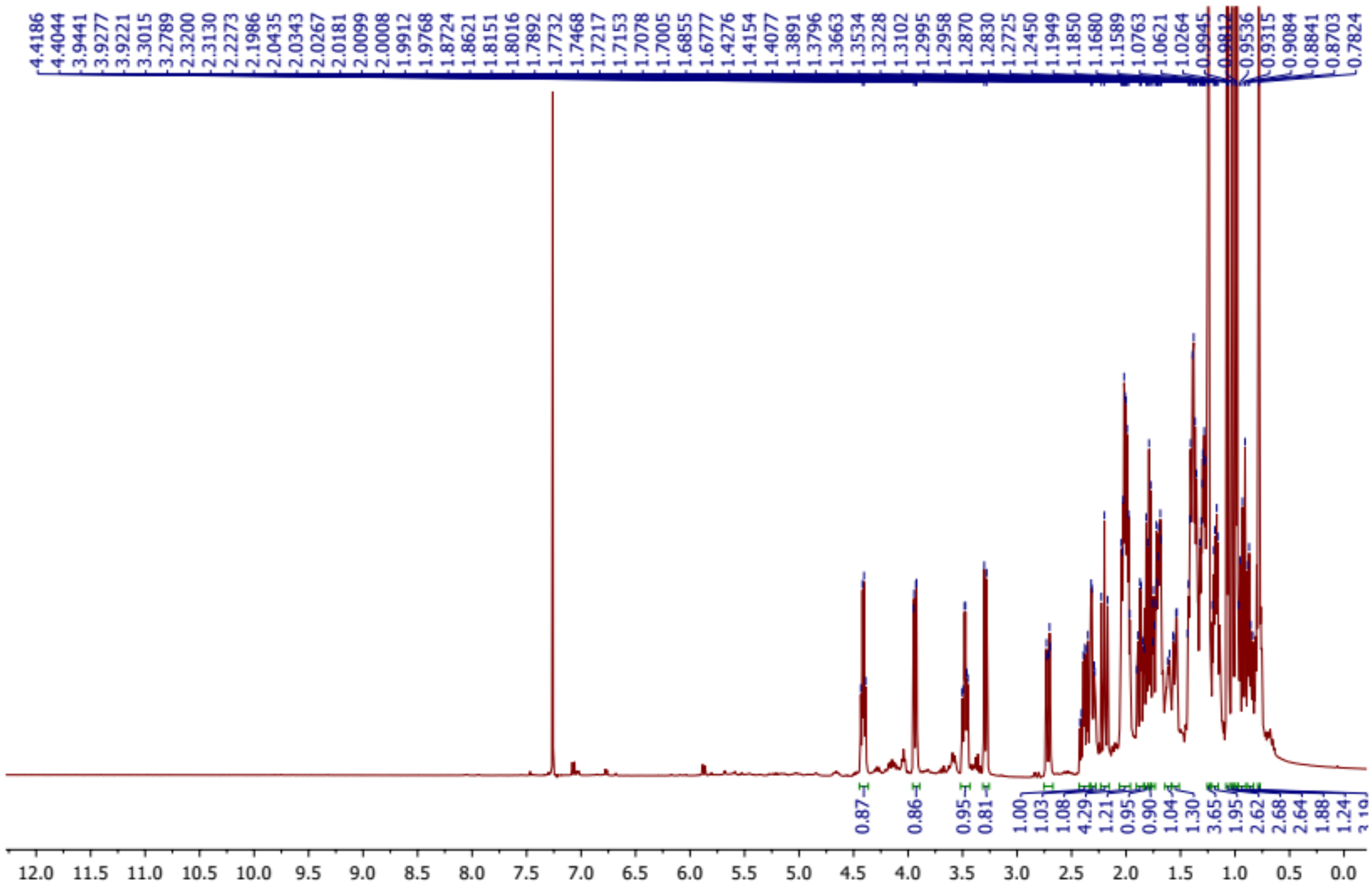

Figure S14. ^{1}H NMR spectrum of compound **3** (500 MHz, $CDCl_3$)

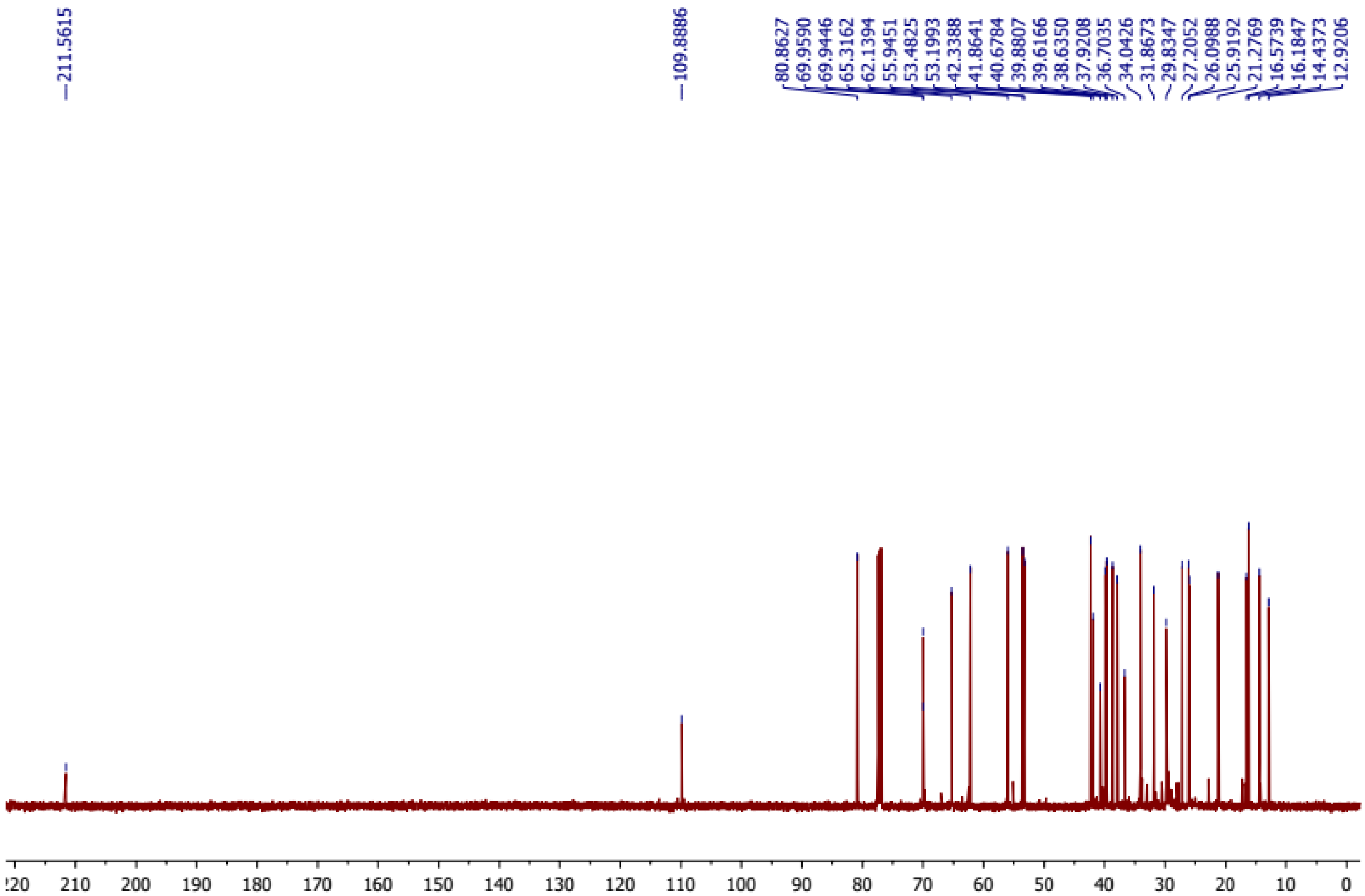

Figure S15. ^{13}C NMR spectrum of compound **3** (125 MHz, $CDCl_3$)

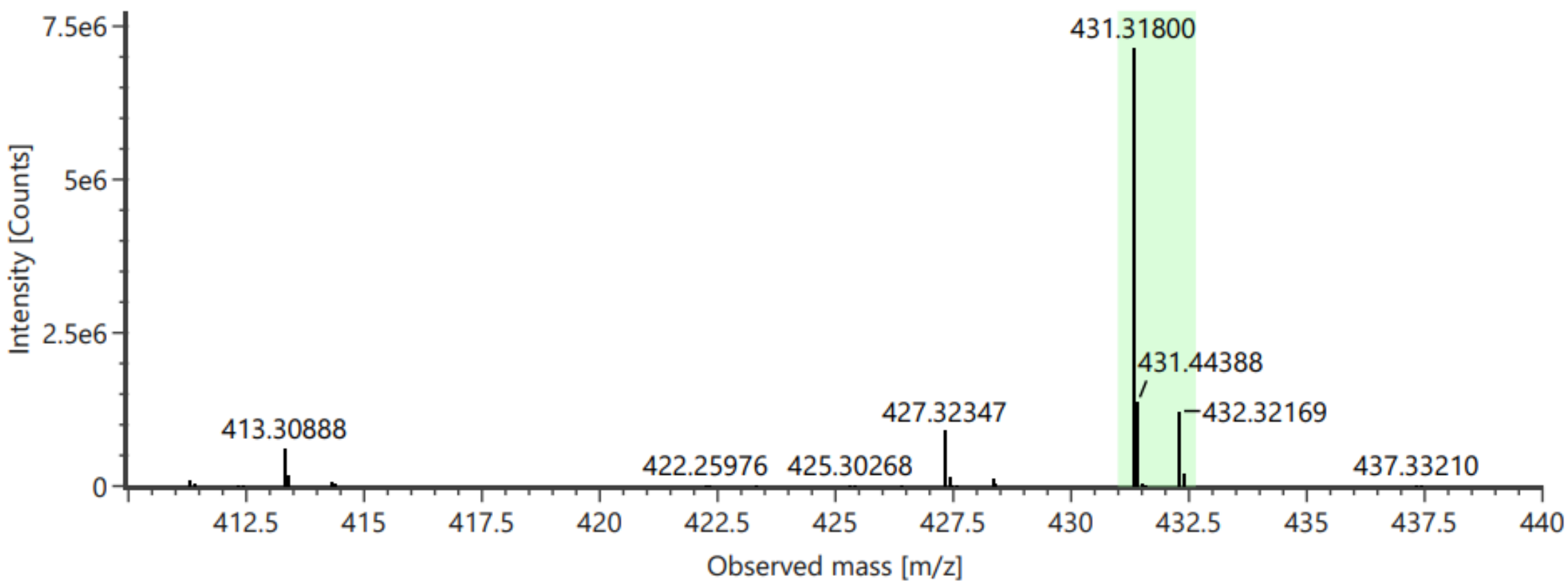

Figure S16. MS spectrum of compound **3**

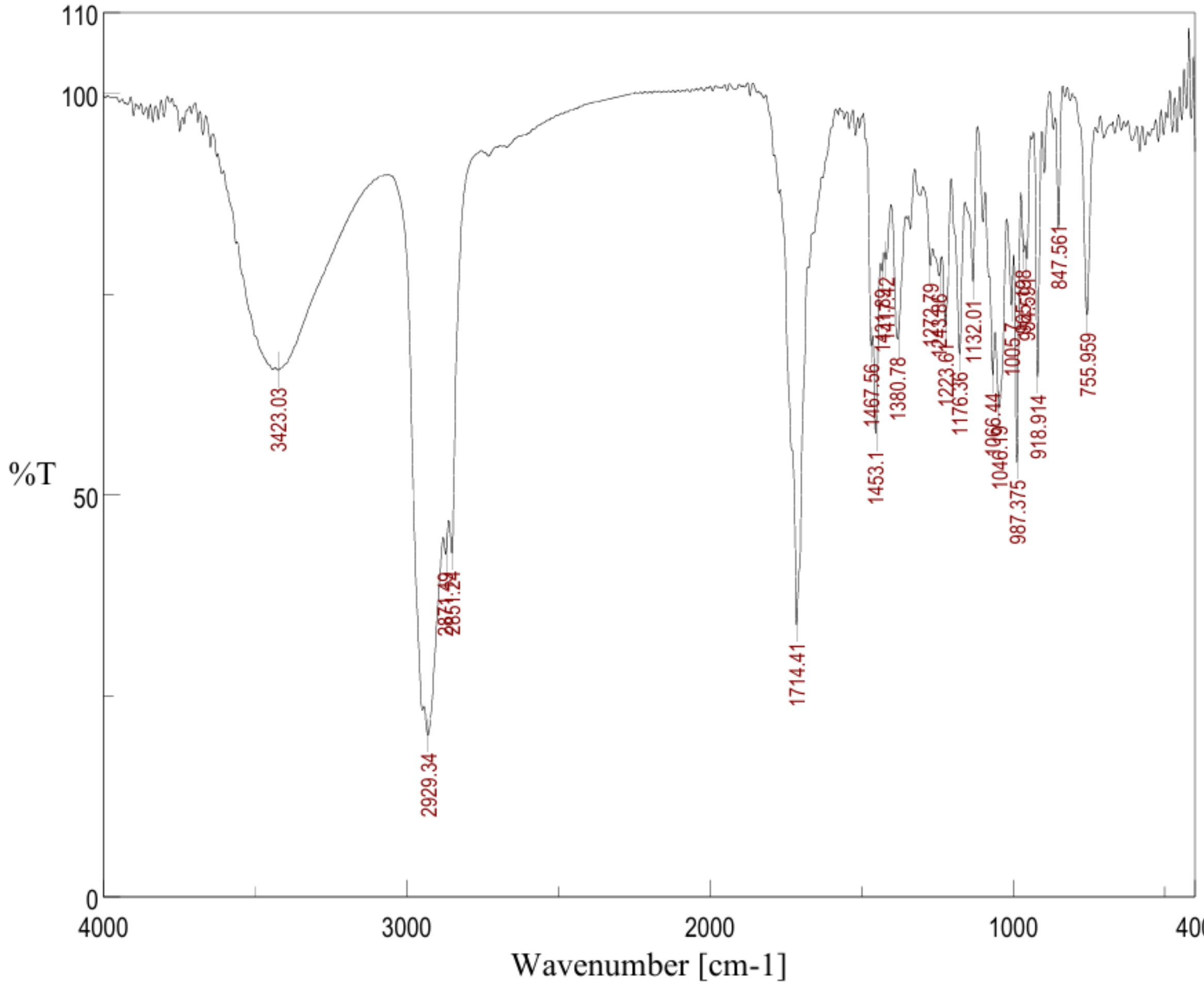

Figure S17. FTIR spectrum of compound **3**

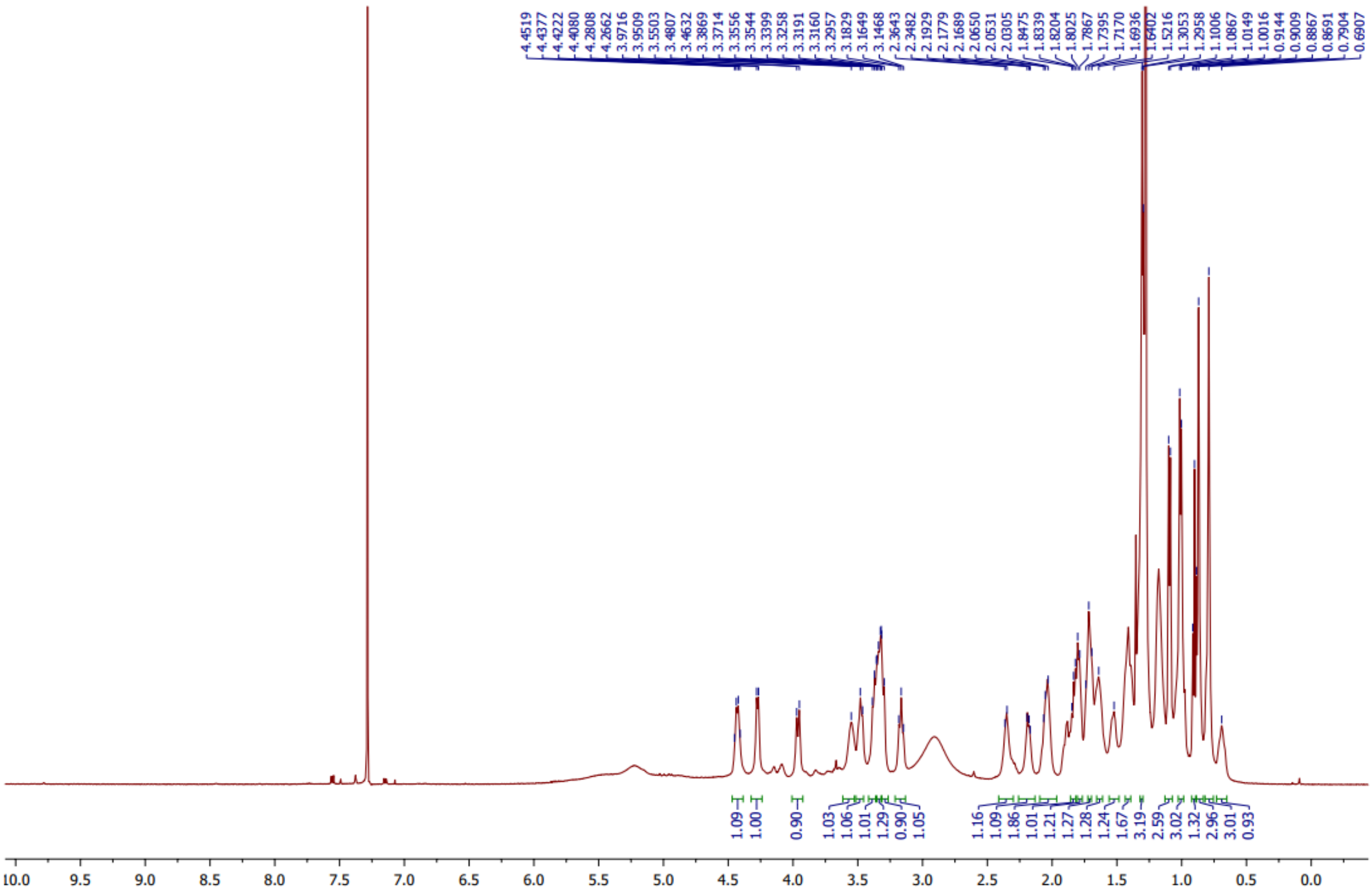

Figure S18. ^{1}H NMR spectrum of compound **4** (500 MHz, $CDCl_3$)

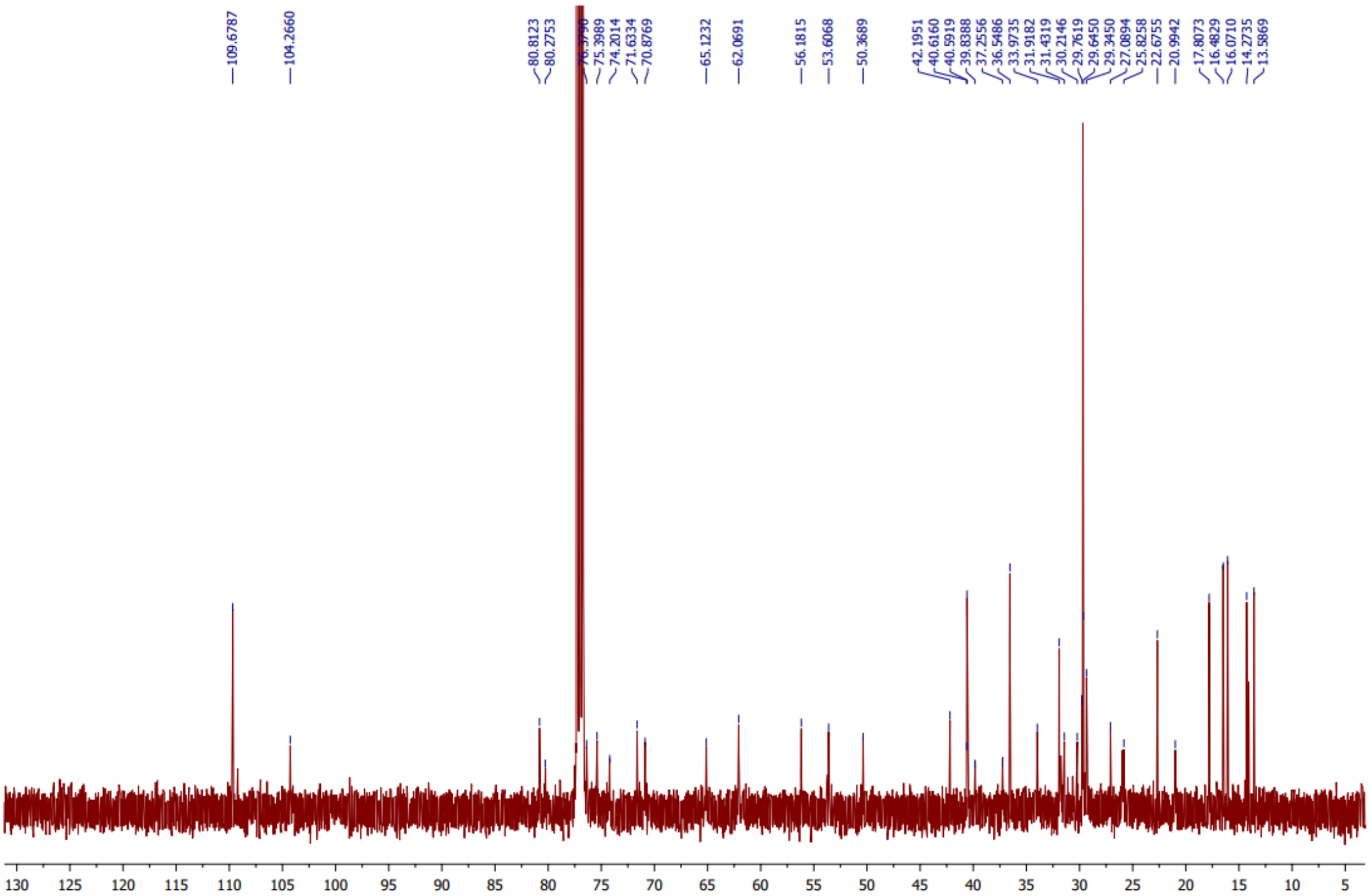

Figure S19. ^{13}C NMR spectrum of compound **4** (125 MHz, $CDCl_3$)

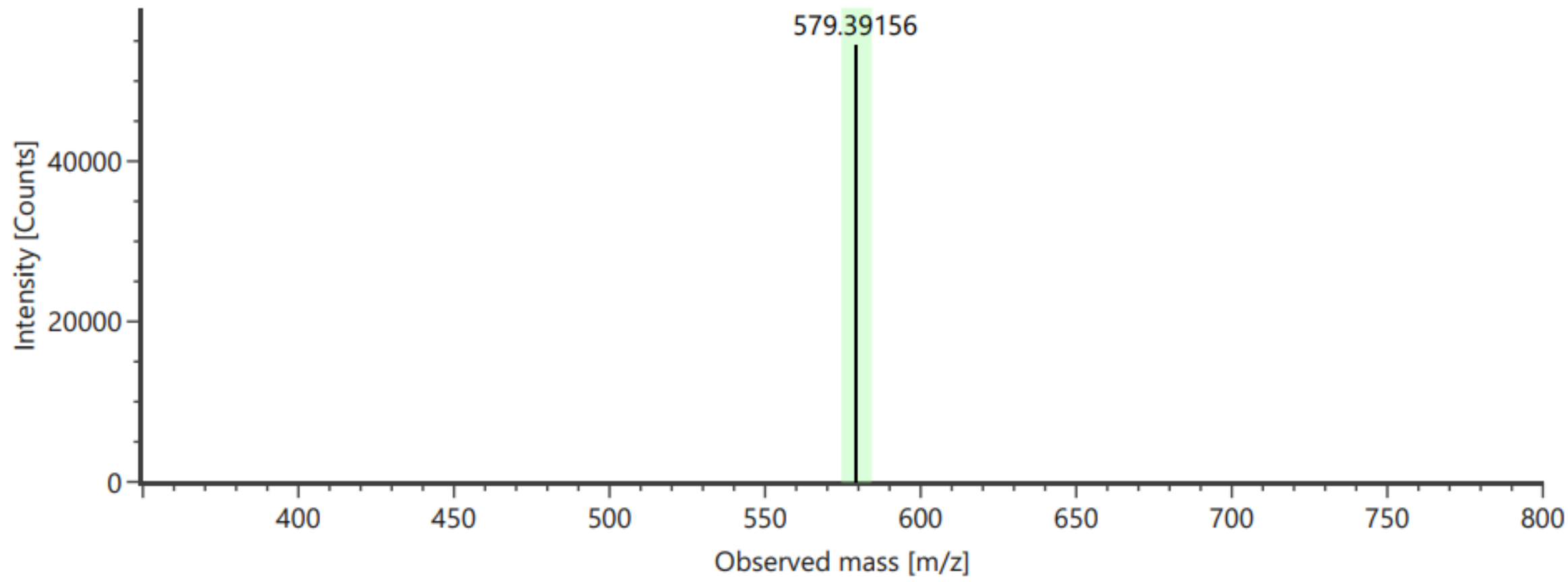

Figure S20. MS spectrum of compound **4**

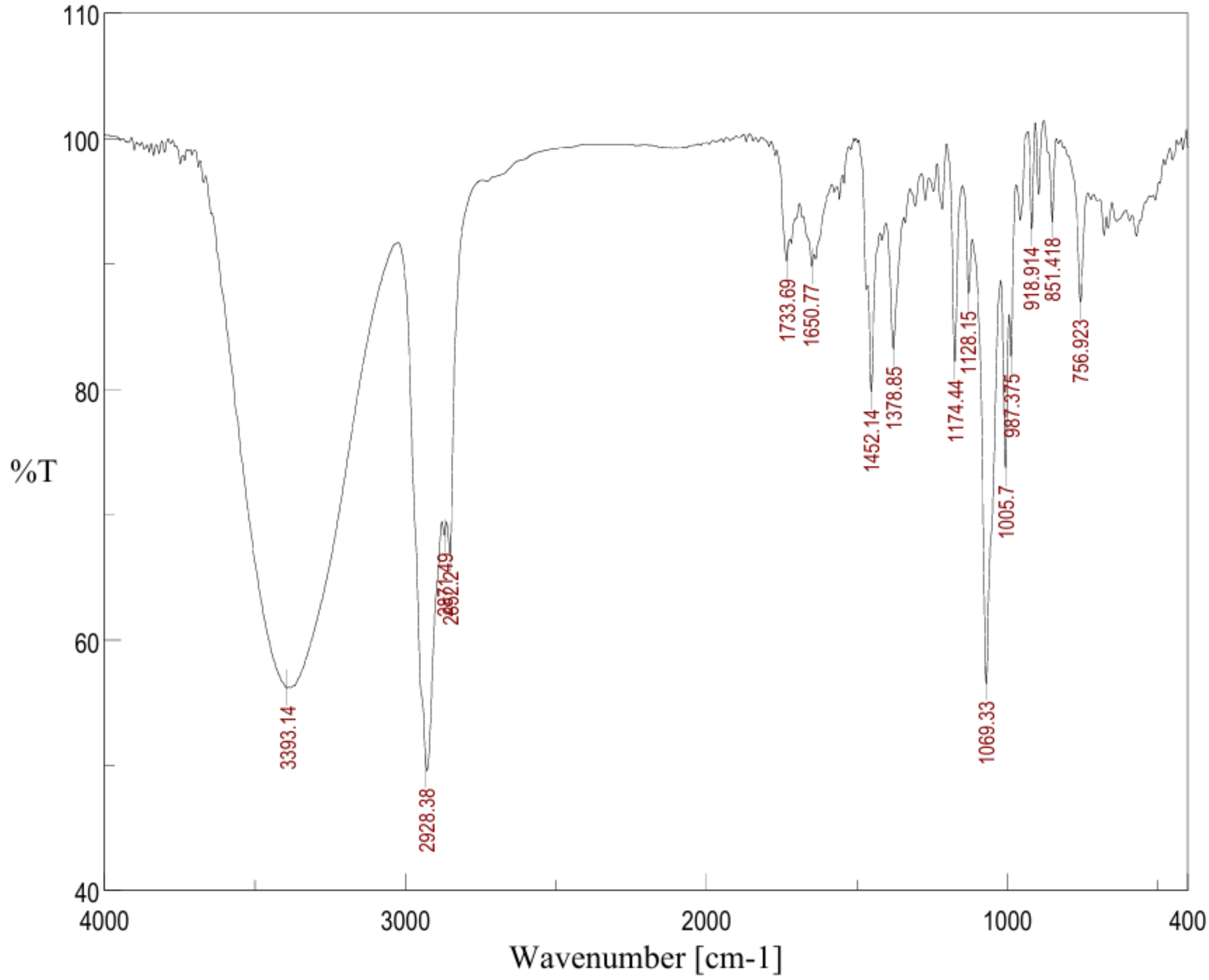

Figure S21. FTIR spectrum of compound **4**

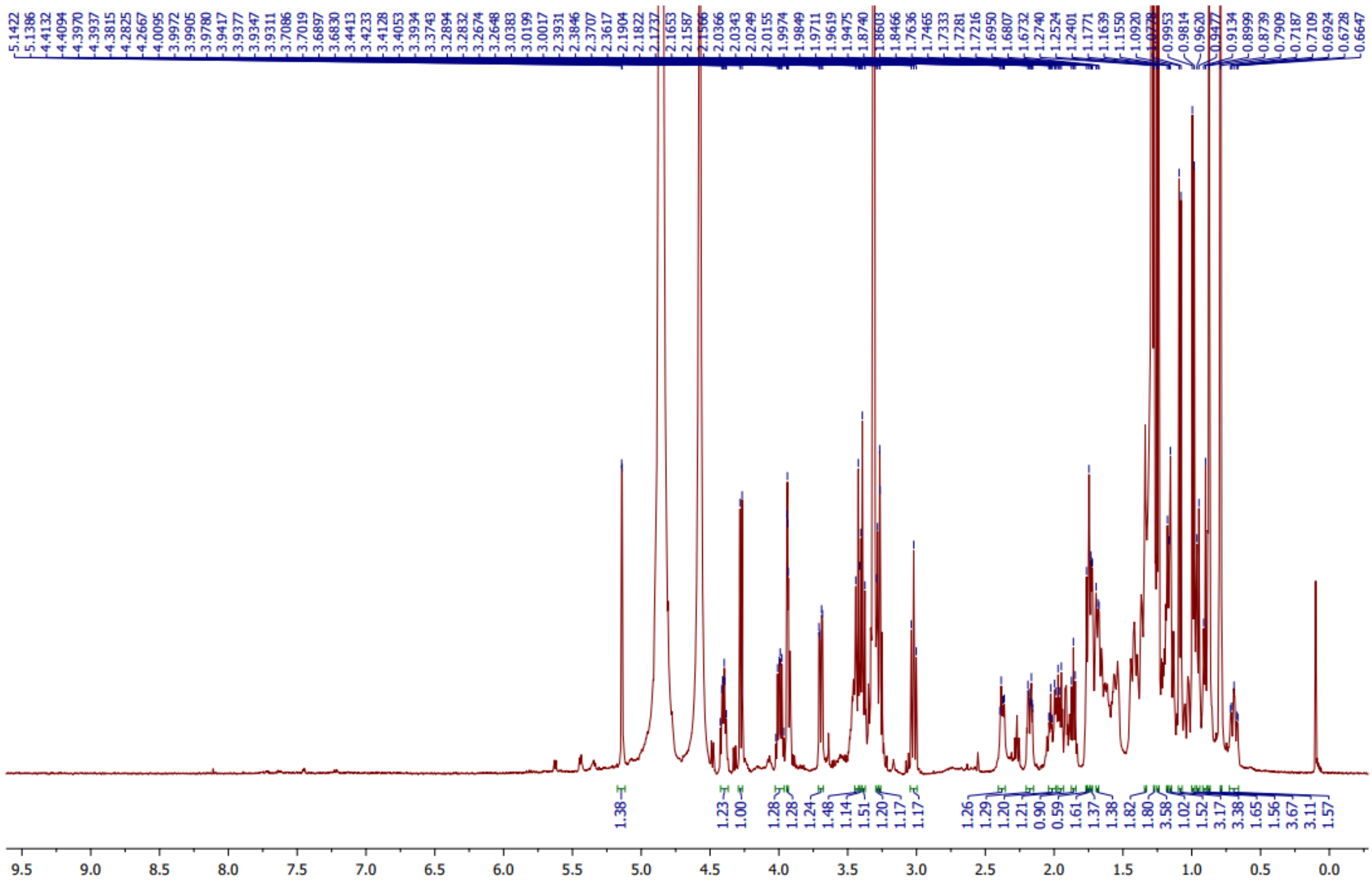

Figure S22. ^{1}H NMR spectrum of compound **5** (500 MHz, CD_3OD)

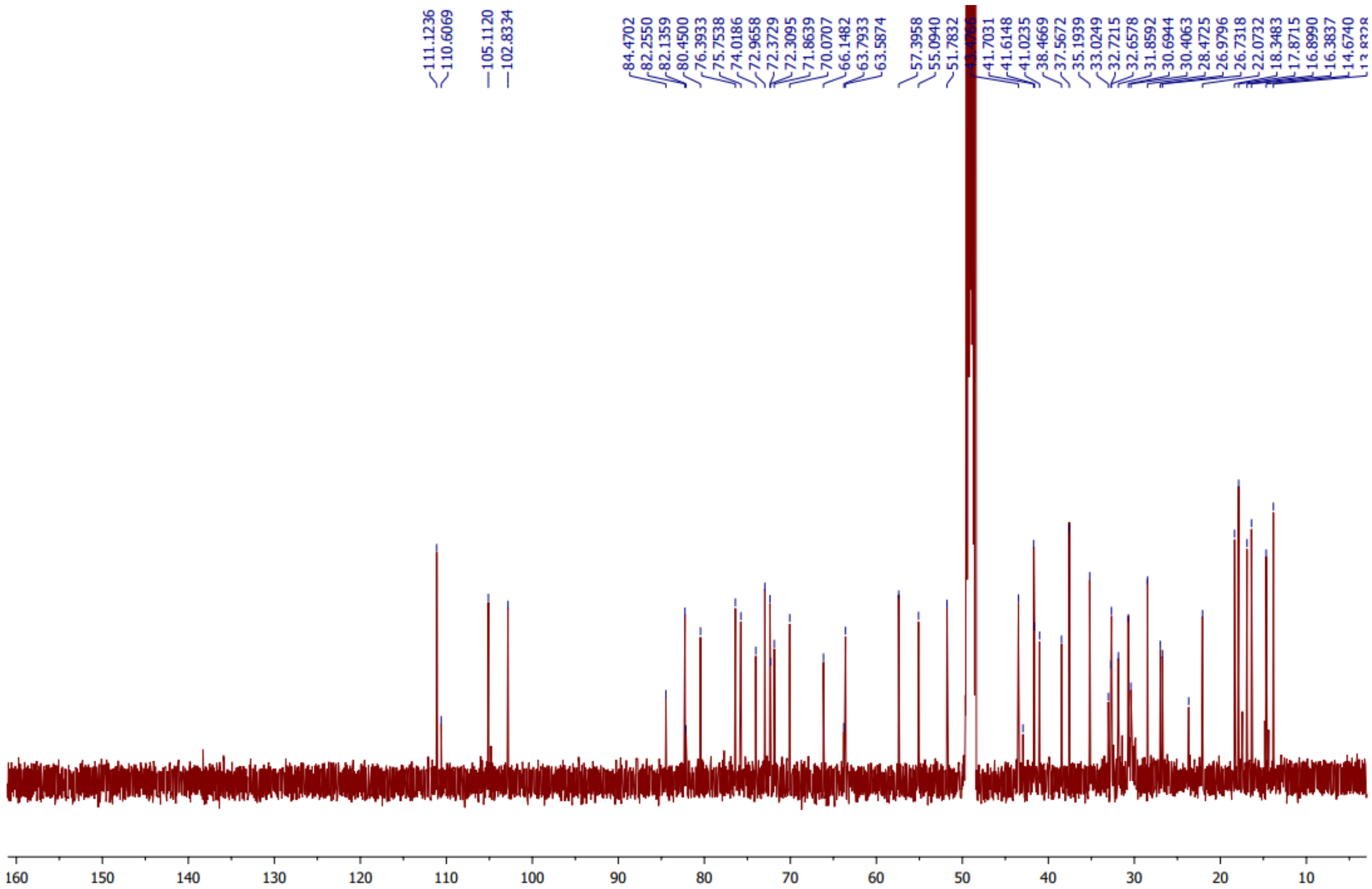

Figure 23. ^{13}C NMR spectrum of compound **5** (125 MHz, CD_3OD)

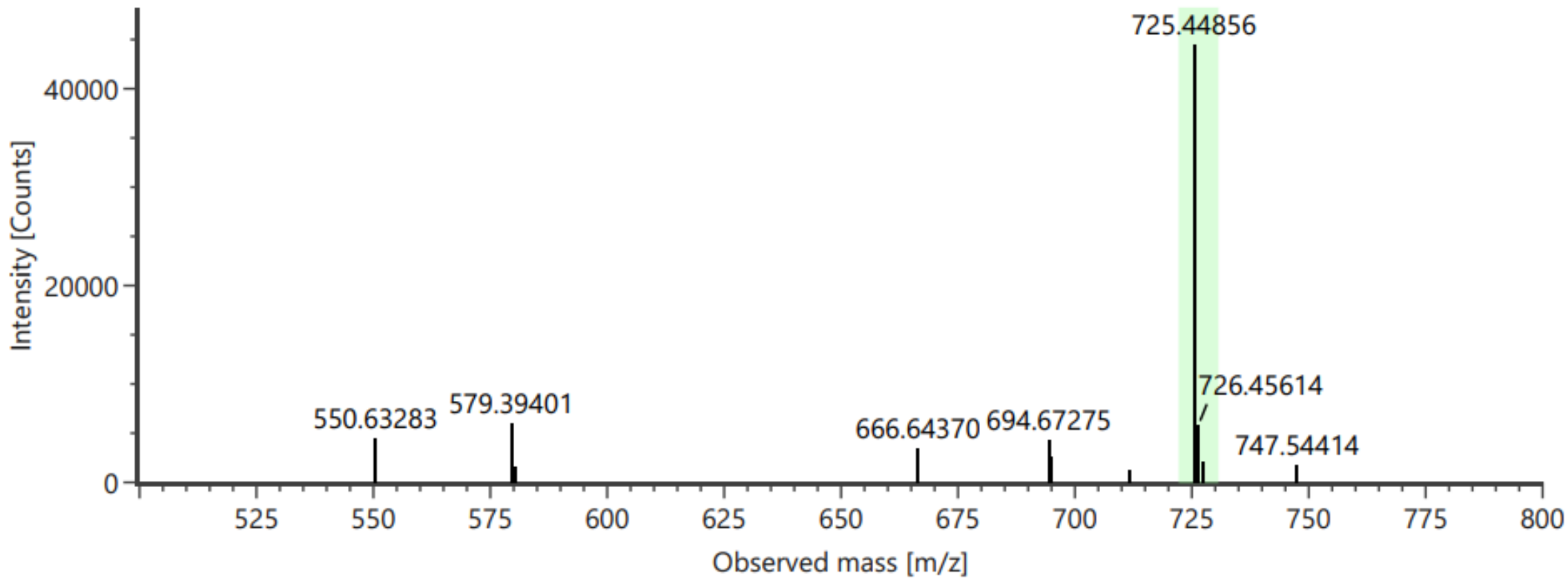

Figure S24. MS spectrum of compound **5**

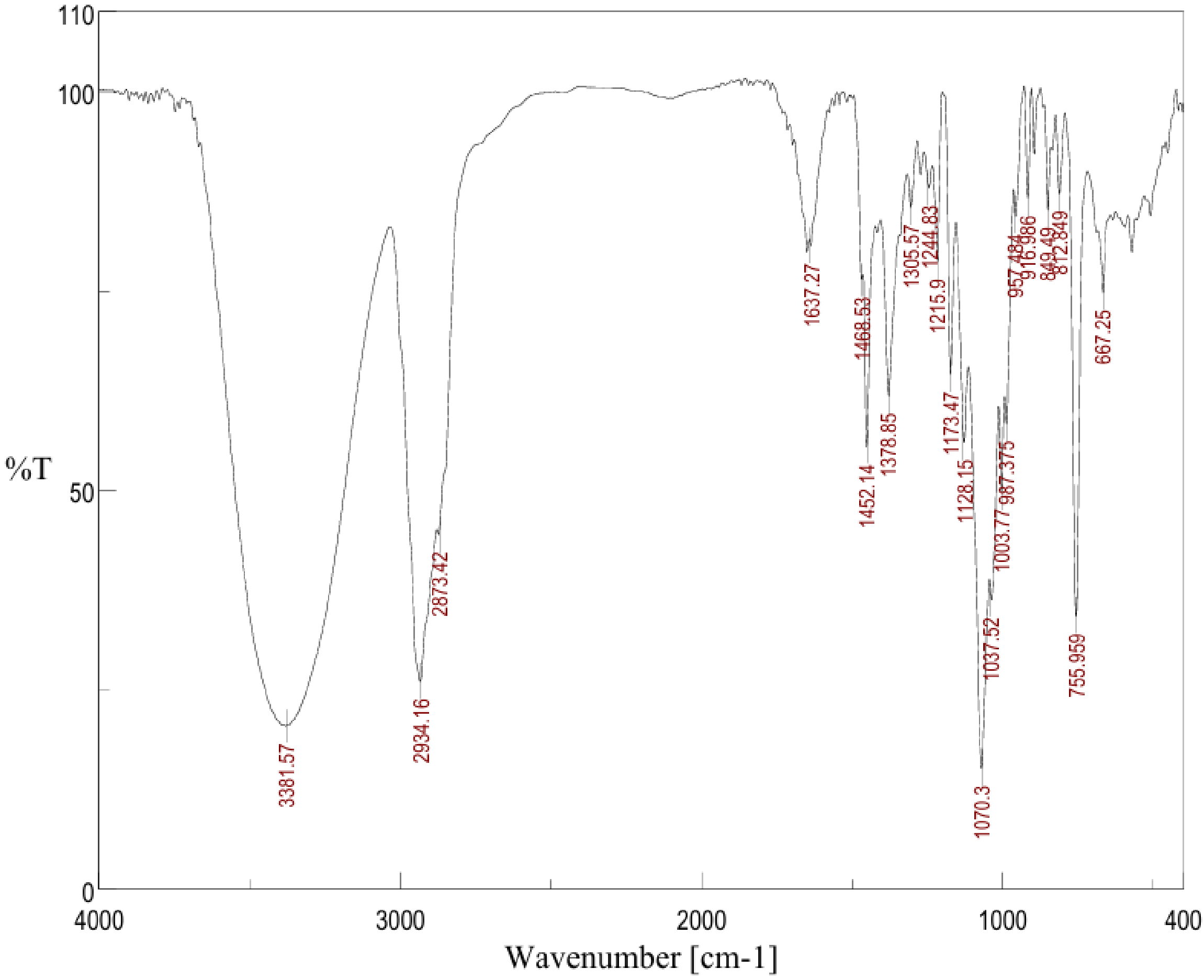

Figure S25. FTIR spectrum of compound **5**

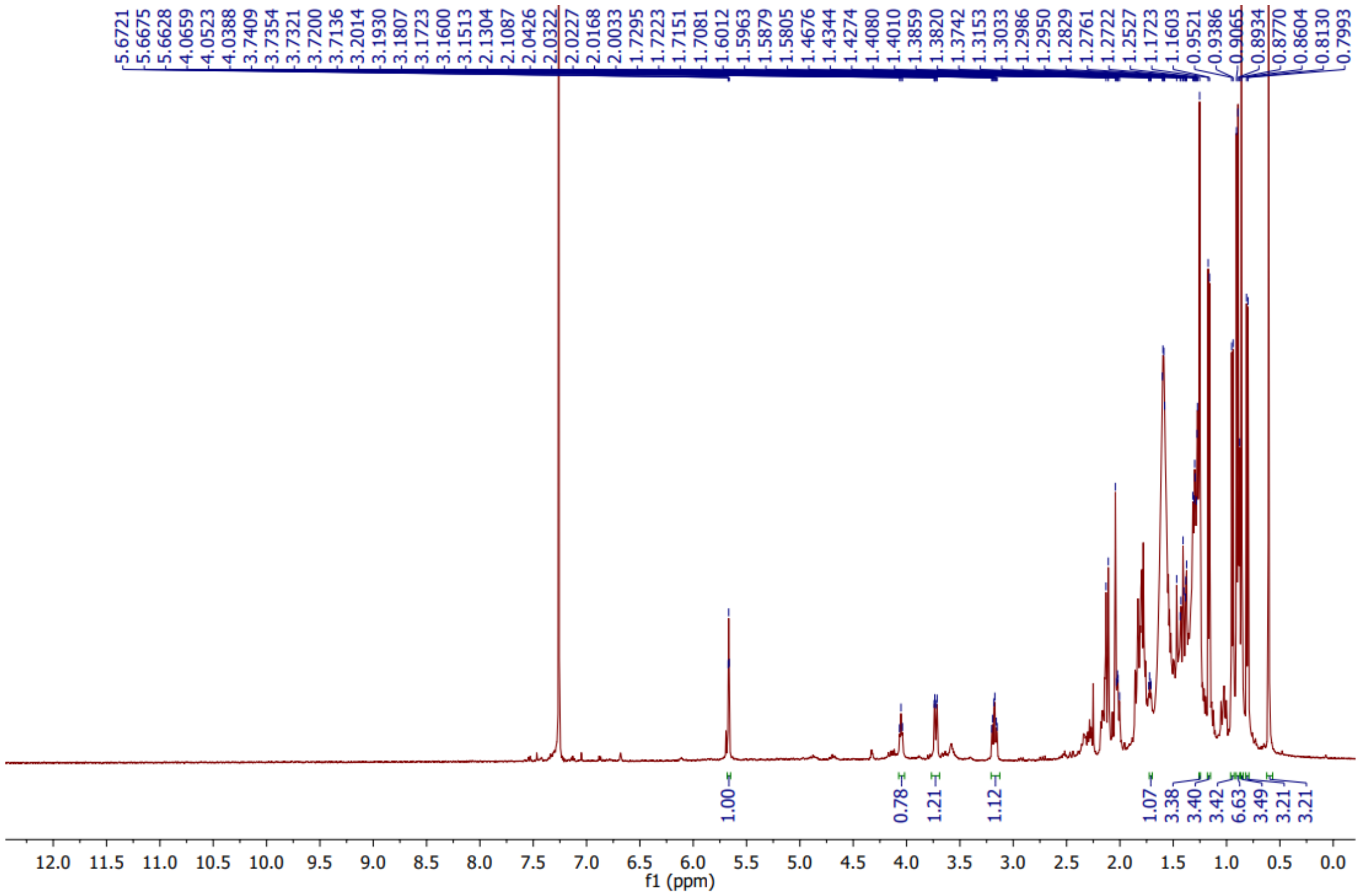

Figure S26. ^{1}H NMR spectrum of compound **6** (500 MHz, $CDCl_3$)

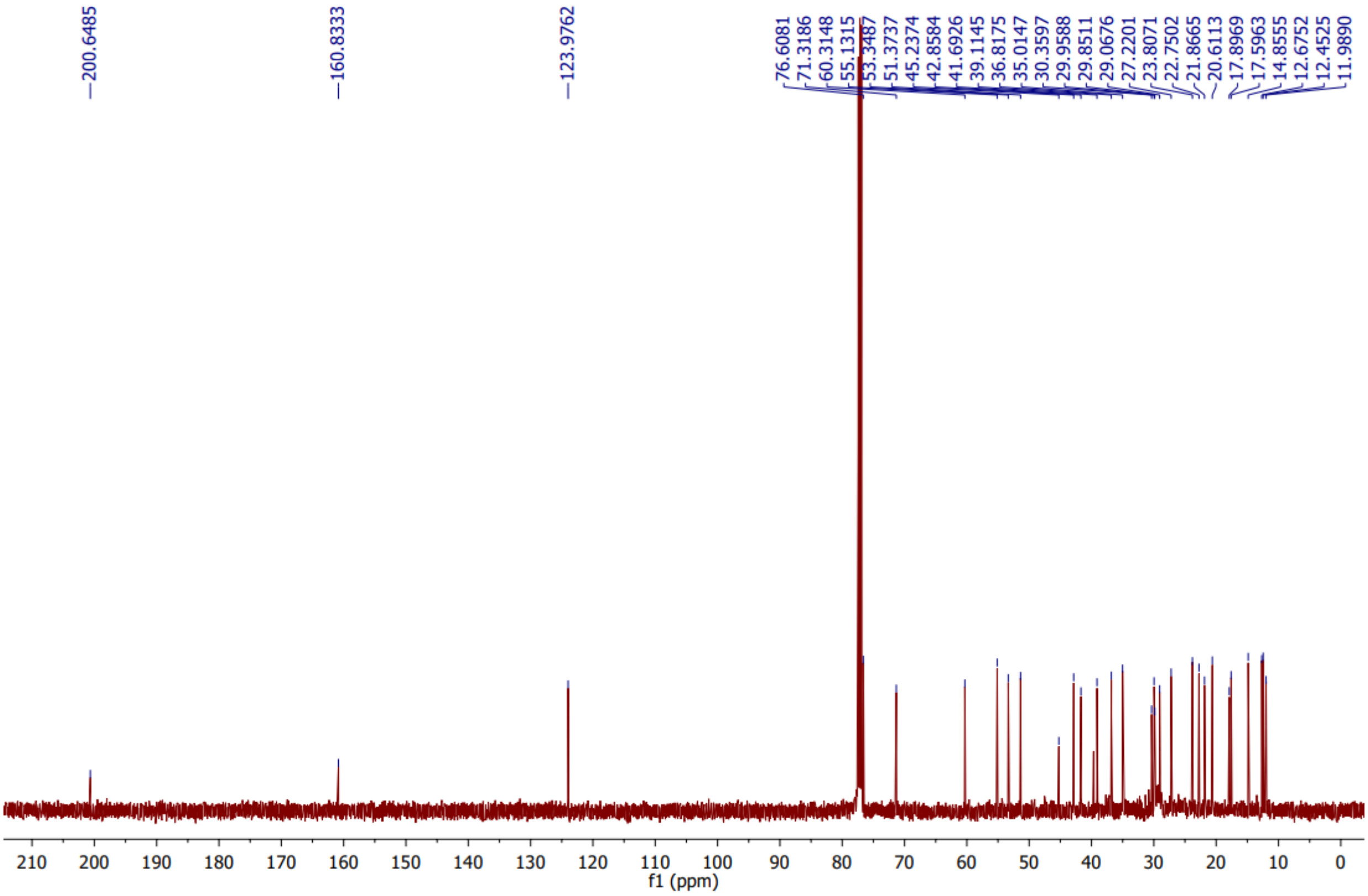

Figure S27. ^{13}C NMR spectrum of compound **6** (125 MHz, $CDCl_3$)

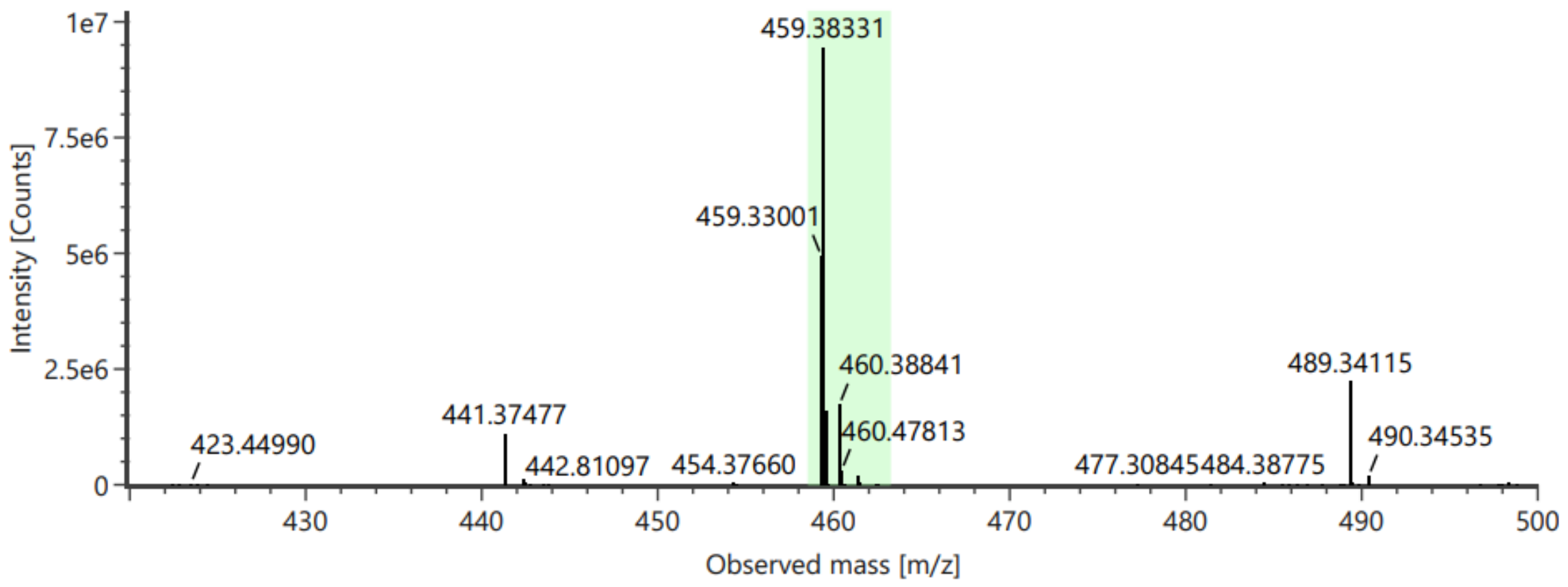

Figure S28. MS spectrum of compound **6**

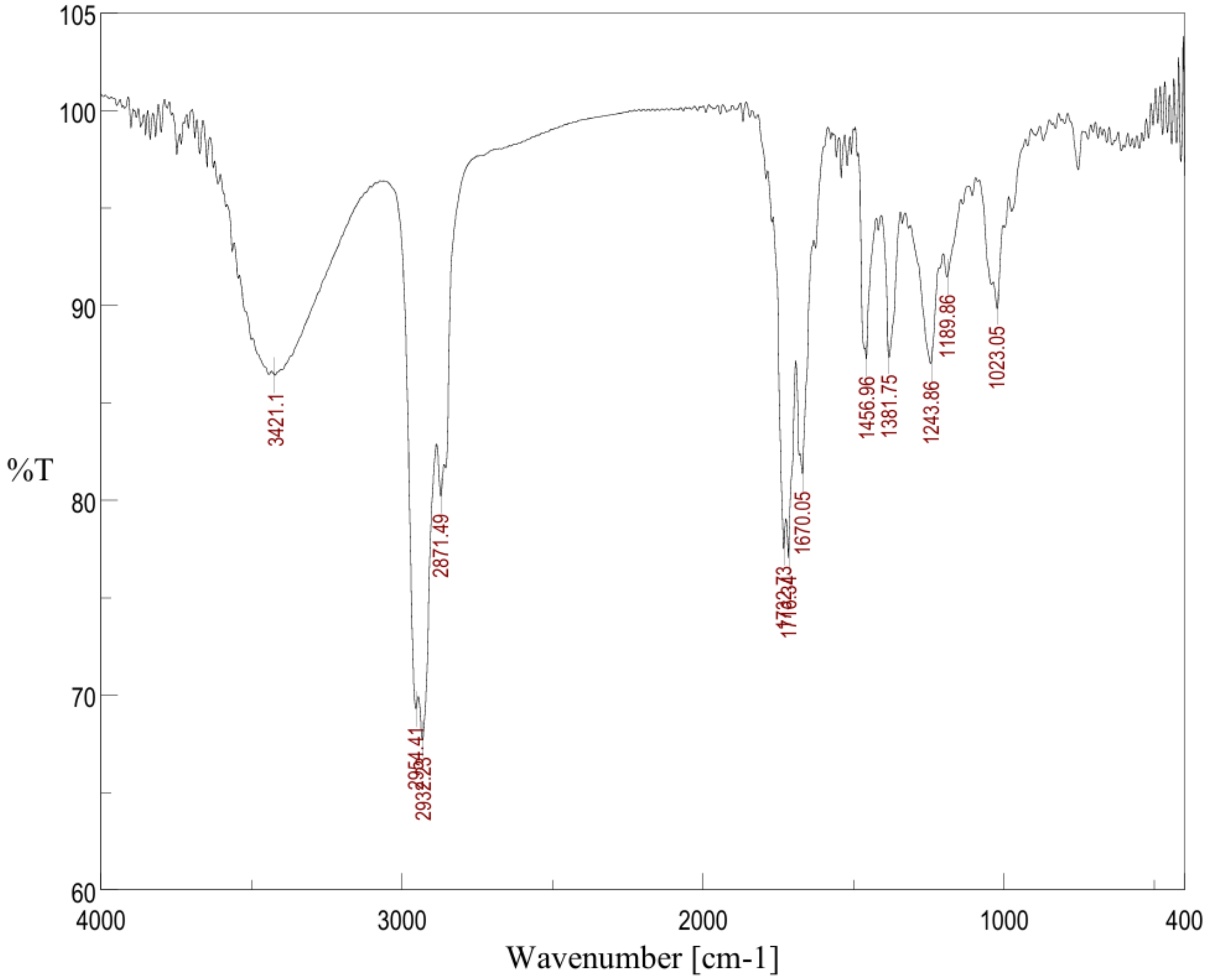

Figure S29. FTIR spectrum of compound **6**

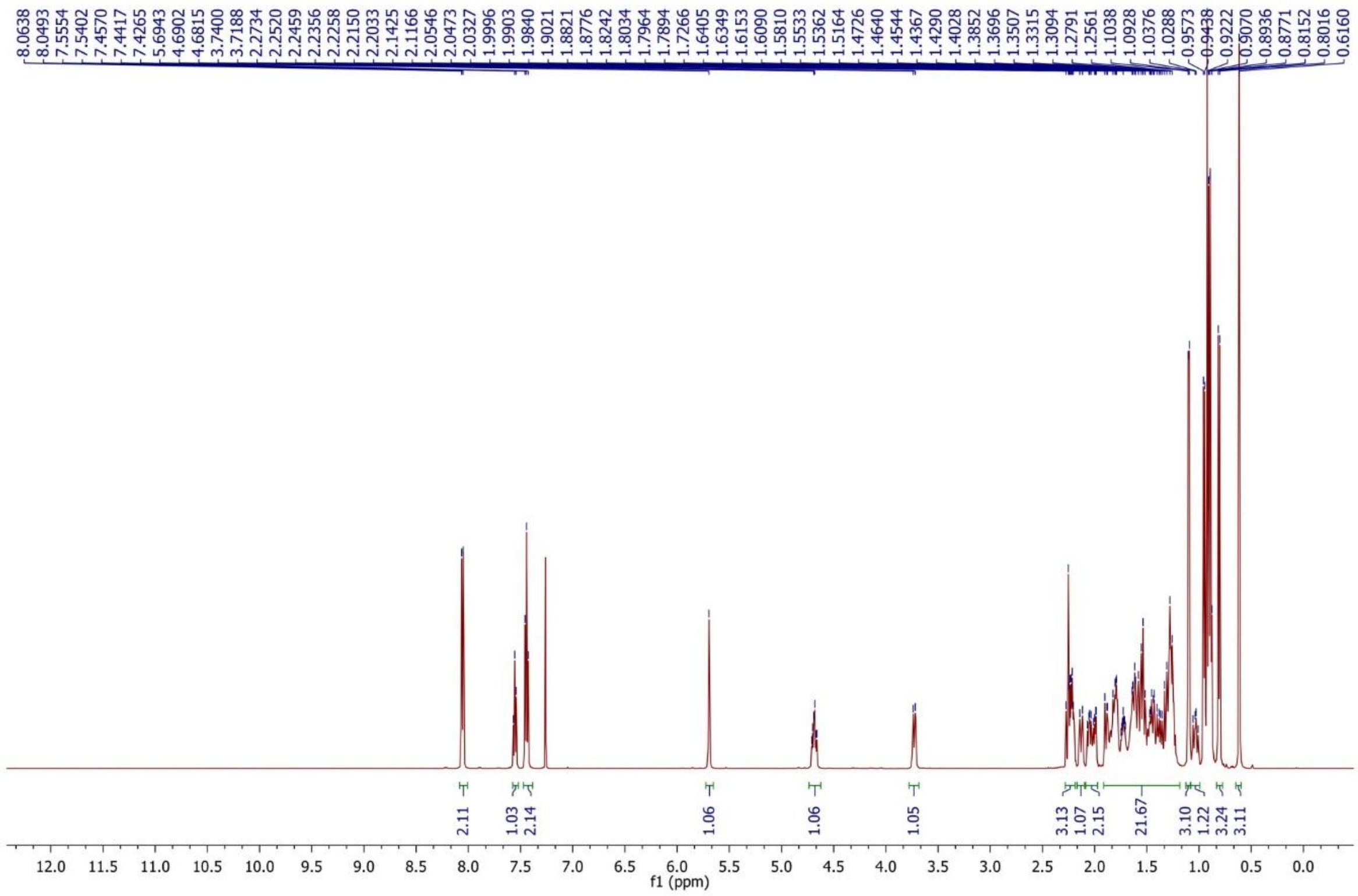

Figure S30. ^{1}H NMR spectrum of compound **7** (500 MHz, $CDCl_3$)

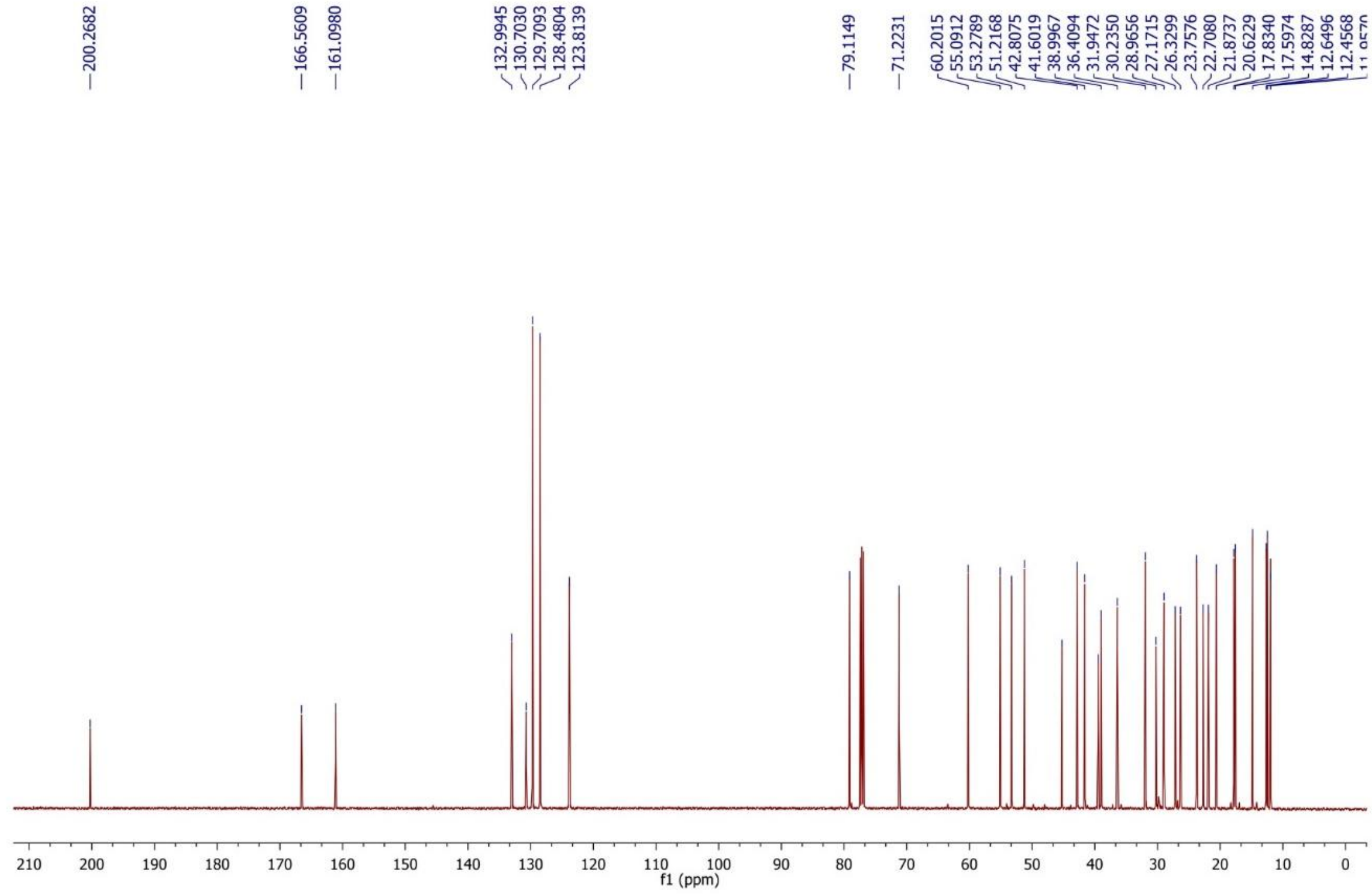

Figure S31. ^{13}C NMR spectrum of compound **7** (125 MHz, $CDCl_3$)

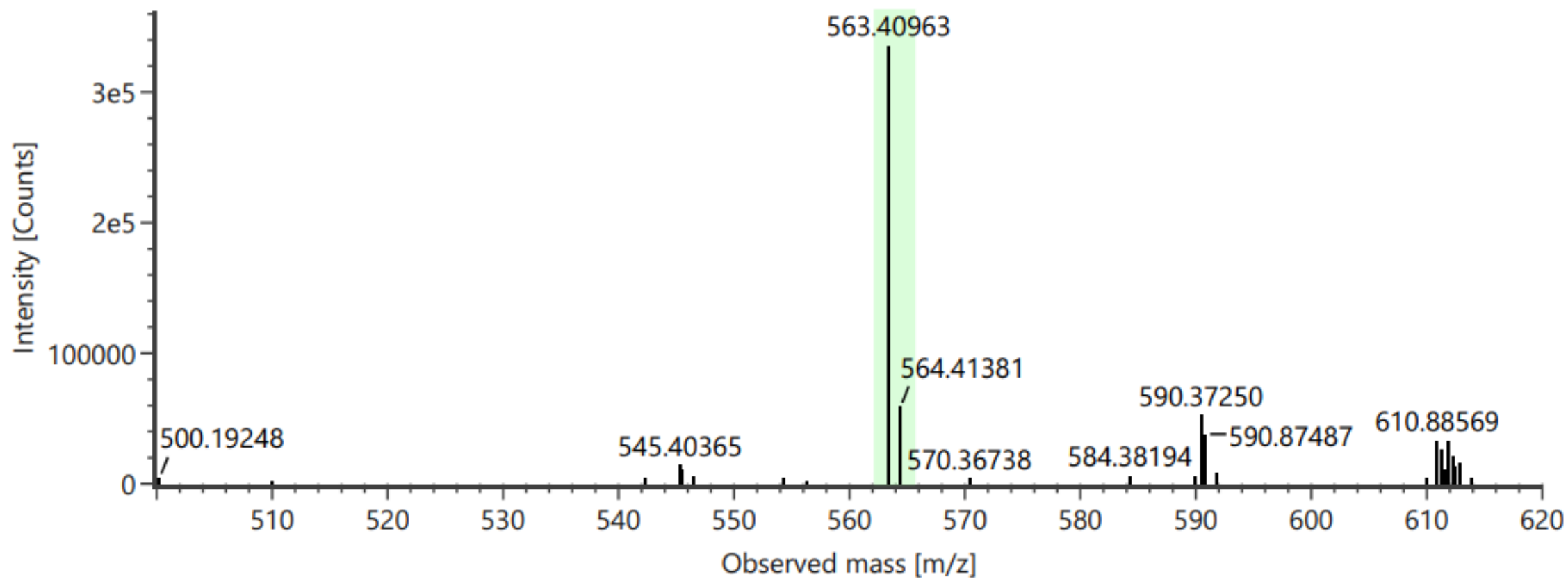

Figure S32. MS spectrum of compound **7**

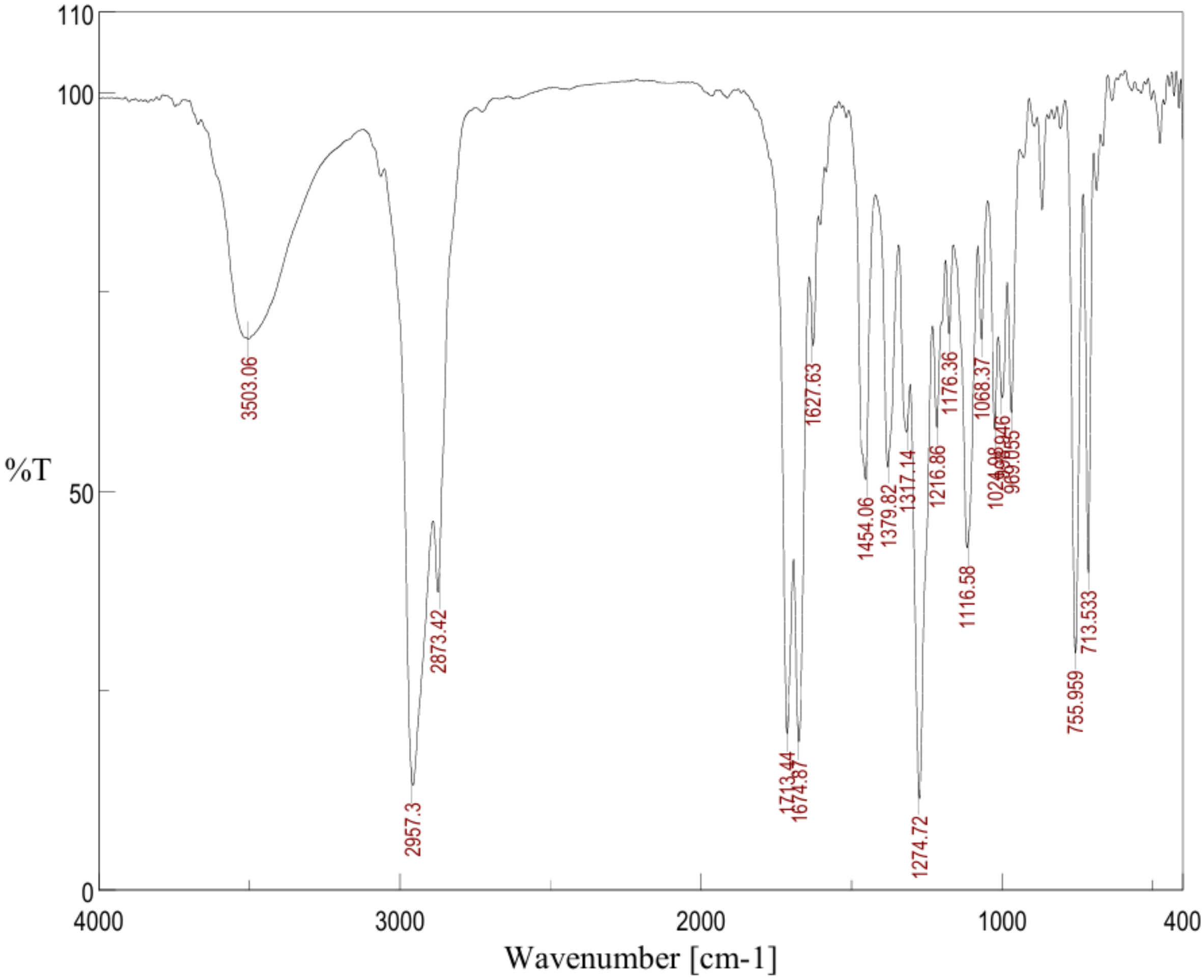

Figure S33. FTIR spectrum of compound **7**

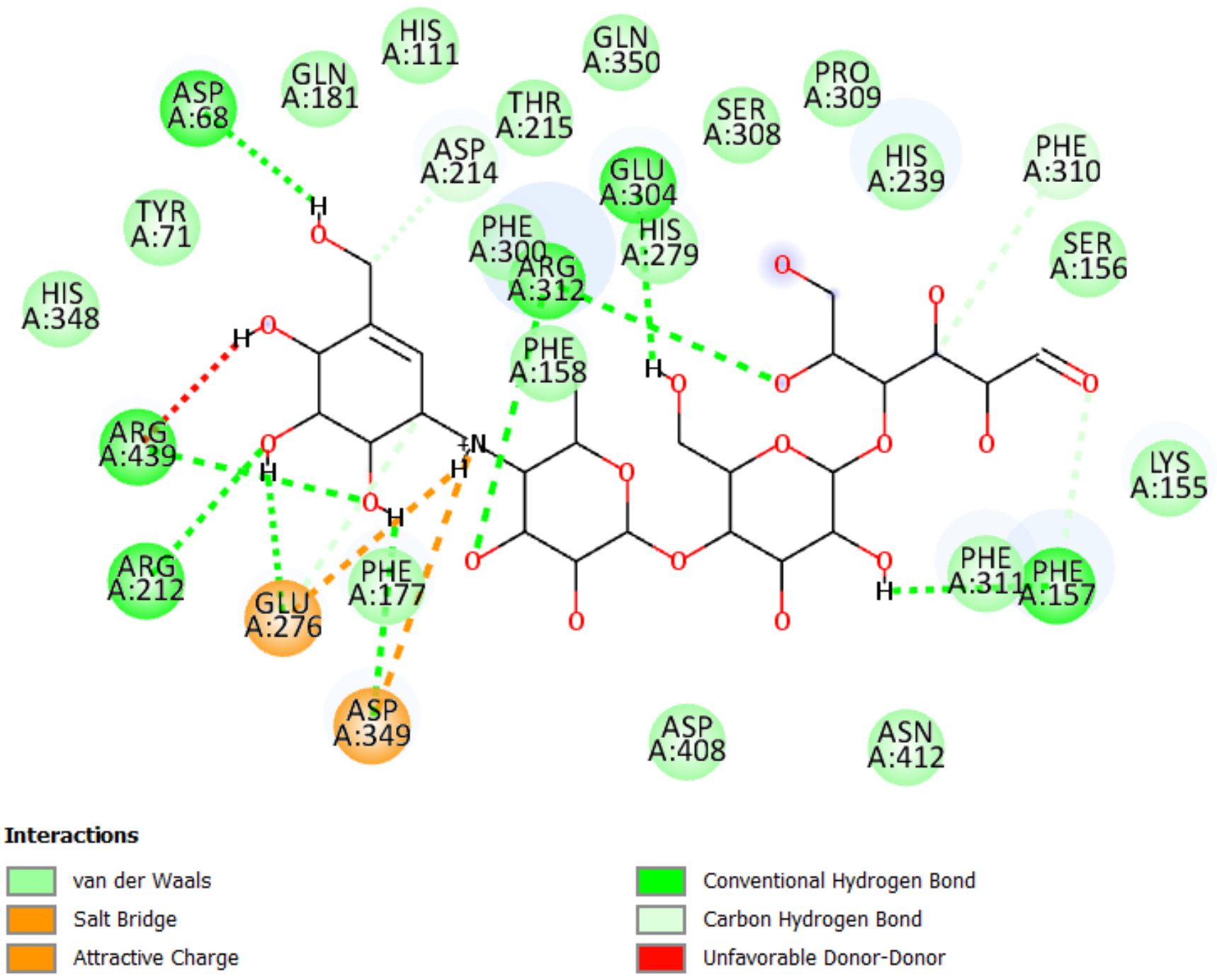

Figure S34. 2D interaction diagrams of *α*-glucosidase in complex with acarbose

Table S2. Summary geometry and Ramachandran evaluation of *α*-glucosidase homology model on MolProbity (V. Chen, W. Arendall, J. Headd, D. Keedy, R. Immormino, G. Kapral, L. Murray, J. Richardson and D. Richardson, Acta Crystallographica Section D, 2009, 66, 12-21.)

				Criteria based on MolProbity
All-Atom Contacts	Clashscore, all atoms:	19.56		34th percentile* (N=1784, all resolutions)
	Clashscore is the number of serious steric overlaps (> 0.4 Å) per 1000 atoms.			
Protein Geometry	Poor rotamers	0	0.00%	Goal: <0.3%
	Favored rotamers	517	99.04%	Goal: >98%
	Ramachandran outliers#	3	0.52%	Goal: <0.05%
	Ramachandran favored	561	96.39%	Goal: >98%
	Rama distribution Z-score	-0.74 ± 0.32		Goal: abs(Z score) < 2
	MolProbity score^	2.03		74th percentile* (N=27675, 0Å - 99Å)

	Cβ deviations >0.25Å	0	0.00%	Goal: 0
	Bad bonds:	90 / 4973	1.81%	Goal: 0%
	Bad angles:	11 / 6736	0.16%	Goal: <0.1%
Peptide Omegas	Cis Prolines:	1 / 28	3.57%	Expected: ≤1 per chain, or ≤5%
Low-resolution Criteria	CaBLAM outliers	9	1.6%	Goal: <1.0%
	CA Geometry outliers	2	0.34%	Goal: <0.5%
Additional validations	Chiral volume outliers	0/677		
	Waters with clashes	0/0	0.00%	

* 100th percentile is the best among structures of comparable resolution; 0th percentile is the worst.

^ MolProbity score combines the clashscore, rotamer, and Ramachandran evaluations into a single score, normalized to be on the same scale as X-ray resolution.

[#] Ramachandran outliers included Thr2, Ile3, and Ala278.
